# Convergence behaviour and Control in Non-Linear Biological Networks

**DOI:** 10.1038/srep09746

**Published:** 2015-06-11

**Authors:** Stefan Karl, Thomas Dandekar

**Affiliations:** 1 Department of Bioinformatics, University of Würzburg, Am Hubland, 97074 Würzburg, Germany

## Abstract

Control of genetic regulatory networks is challenging to define and quantify. Previous control centrality metrics, which aim to capture the ability of individual nodes to control the system, have been found to suffer from plausibility and applicability problems. Here we present a new approach to control centrality based on network convergence behaviour, implemented as an extension of our genetic regulatory network simulation framework Jimena ( http://stefan-karl.de/jimena). We distinguish three types of network control, and show how these mathematical concepts correspond to experimentally verified node functions and signalling pathways in immunity and cell differentiation: Total control centrality quantifies the impact of node mutations and identifies potential pharmacological targets such as genes involved in oncogenesis (e.g. zinc finger protein GLI2 or bone morphogenetic proteins in chondrocytes). Dynamic control centrality describes relaying functions as observed in signalling cascades (e.g. src kinase or Jak/Stat pathways). Value control centrality measures the direct influence of the value of the node on the network (e.g. Indian hedgehog as an essential regulator of proliferation in chondrocytes). Surveying random scale-free networks and biological networks, we find that control of the network resides in few high degree driver nodes and networks can be controlled best if they are sparsely connected.

Since the advent of high throughput gene expression profiling, ever more aspects of cell phenotypes in humans^1^,^2^,^3^,^4^, other animals^5^, fungi^6^,^7^, plants^8^,^9^,^10^ and bacteria^11^,^12^ can be modelled by semi-quantitative regulatory networks which describe genetic or metabolic regulatory processes by mathematical functions.

In these networks, molecules such as genes, proteins and hormones are represented by abstract nodes 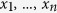, whose values 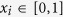 correspond to the current activity of the nodes, e.g. the gene expression level, the concentration of a hormone, or the activation of a protein by post-translational modifications. In simple, linear models, the changes of these values over time are governed by differential equations of the form 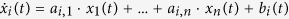
13 where 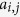 are numerical constants describing kinetic properties of the interaction between nodes 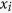 and 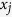, and 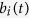 is an optional perturbation component. Nodes appearing in the differential equation of another node influence the value of that node.

Although kinetic data on interactions is often sparse^14^,^15^, non-linear elements like high degree polynomial functions or exponential functions in the differential equation have proven useful in modelling the dynamics of biological networks, and are implemented in mathematical templates such as the exponential standardised qualitative dynamical systems (SQDS) method^16^,^17^, and the HillCube high degree polynomial interpolation of Boolean functions^18^. For example, these non-linear functions allow for the modelling of switch-like characteristics where the behaviour of a target node changes sharply if the value of an influencing node rises beyond a certain threshold. Networks in which at least one non-linear component appears are called non-linear networks.

To gain new biological insights, understanding the functions of nodes, interactions and pathways in the dynamic systems arising from these differential equations is crucial (we are not dealing here with structural or topological measures of centrality such as closeness, diameter or betweenness).

Previous research has established the concept of controllability^13^,^19^, i.e. the ability of a subset of the nodes to control the behaviour of the network. In linear networks, it was proposed to consider the minimal number of driver nodes which allow for the steering of the network to any desired state in finite time (Kalman′s controllability rank condition)^20^. Since determining the minimal number of driver nodes N_d_ is not computationally feasible for most networks, the concept was modified to structural controllability^13^ which efficiently approximates N_d_ at the cost of disregarding numerical network parameters such as relative influence strengths. According to that definition, in both random and real networks, influential driver nodes tend to have only few connections to other network nodes. Around 80%^13^ of the network nodes are then necessary to drive a biological system, which has been criticised for contradicting experimental findings suggesting that a small number of nodes dominate network behaviour^21^,^22^.

While controllability describes a property of the network as a whole, different definitions of control centrality aim to assess the influence a single node has on the behaviour of the network.

A previous concept^23^ has been derived from structural controllability, but suffers from the same problems in so far as it is only applicable in linear networks and disregards any numerical parameters in the differential equations, which are crucial for the behaviour of the network.

An alternative approach to controllability^19^ investigated how networks can be controlled by subsets of the nodes, whose activity can be reduced at will at any given time, but not increased. While this definition is applicable in non-linear networks, the limitation to deactivating manipulations lacks biological plausibility since nodes in biological systems can be subject to strong activation as well as inhibition, as commonly observed for overexpressed oncogenes or targets of pharmacological interventions. A control centrality metric called “participation rate” Cornelius *et al.* derive^19^ from their notion of controllability inherits these plausibility problems.

Here we present a novel approach to quantify control centrality which is applicable in linear as well as non-linear networks. Based on network convergence, it does not disregard numerical parameters in the differential equations, thus capturing the whole dynamic behaviour of the system. In all these instances, time changes and convergence behaviour are critical. This includes non-linear components (e.g. Map kinase, death receptor activation) as well as non-linear systems effects (e.g. apoptosis avalanche, cell cycle feedback loops). To our knowledge, control centralities of the connections between the nodes can be quantified for the first time, enabling new insights into signalling pathways. In contrast to previous concepts, we distinguish different types of network control, elucidate the potential impact of genetic manipulations or pharmacological interventions in networks and demonstrate that our metrics are in accordance with experimental findings as shown for networks from cell differentiation^24^,^25^, proliferation control^2^ and immunity^1^,^26^,^27^,^28^. To help application of our measures to other networks, we provide a Java implementation of our concepts including a graphical user interface and the underlying code (see http://stefan-karl.de/jimena/, http://www.bioinfo.biozentrum.uni-wuerzburg.de/computing/jimena-c) together with a tutorial and application examples according to the results presented here.

## Methods

### Convergence and network comparisons

**Figure 1:**
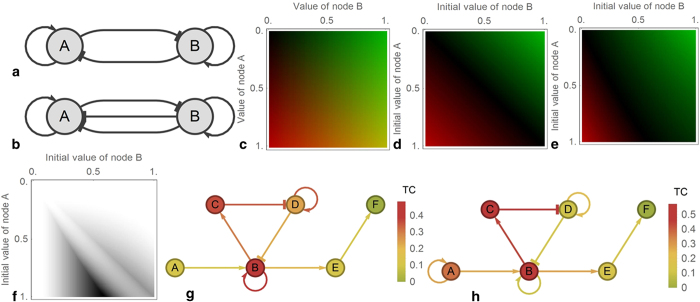
Overview of convergence behaviour and centrality. (**a**) Very often, a simplified graphical representation of the complex differential equations forming a dynamic system can be drawn. In those network graphs, normal arrows usually represent activating influences in the system, and T-shaped arrows represent inhibiting influences. This figure depicts a BooleCube regulatory network N_1_ with two mutually inhibiting nodes A and B. Both nodes hold a value between 0 and 1 representing their activation level, which corresponds for example to the gene expression level in a biological context. (**b**) In network N_2_ the strength of the inhibition B → A has been doubled. (**c**) Colour scheme encoding the values of A (y-axis) and B (x-axis) into a unique colour. Pure green, for example, represents the state A = 0 and B = 1. (**d**) Convergence vectors of the network N_1_ in the colour scheme 1c. Either A, B or none of the nodes are activated (**e**) Convergence of the network N_2_. B is noticeably stronger than A, leading to an expansion of the green area. (**f**) Mean squared difference of the convergence vectors from 1d and 1e (white-0 to black-1). Changes in network behaviour between the two networks are most prominent (i.e. black), if node A is fully activated and node B’s activity is intermediate. (**g**) Total centrality in an artificial network (green-yellow-red scheme, SQDS network). All nodes except for node F influence the behaviour of the network if they are mutated. (**h**) Adding a loop to A enables it to hold its state, greatly increasing its influence.

To really understand control and behaviour in non-linear networks, consideration of network dynamics over time is essential. Mathematically, a time-continuous regulatory network with nodes 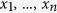 is defined by differential equations 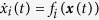 where 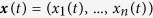 is the state vector of the network at time index 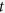. All states 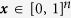 form the state space of the network. Each function 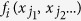 potentially defines multiple connections, i.e. influences of the nodes 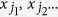 on the node 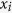 which are usually depicted as arrows in network graphs (Fig. 1a).

We define the convergence difference of two networks with the same nodes as


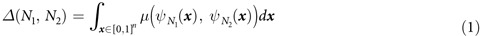


where μ is the mean squared difference and 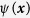 is the state the network 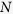 converges to starting from the initial vector 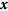 (Supplementary Methods 1.1).

For example, in a gene expression network, the convergence function 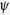 equals letting the system settle to a steady state, where no more changes in gene expression levels occur without new external stimuli. N_1_ and N_2_ could be two intracellular genetic regulatory networks, where N_1_ is the wild-type system, and N_2_ models the nonsense mutation of a gene. The convergence difference function 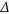 then quantifies how much the steady states of the two dynamic systems differ, if both systems are started from the same initial state 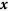. If substantial differences occur for many initial states, the mutation affects the behaviour of the regulatory network.

The concept is explained in Fig. 1. The convergence functions 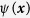 of two small artificial networks (Fig. 1a,b) are plotted in Fig. 1d-1e using the colour scheme from Fig. 1c. The mean squared difference 
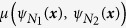
 of the two networks for initial vectors 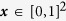 is given in Fig. 1f, and integration over this plot yields the convergence difference 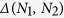.

The convergence difference can also be used as a general measure for the similarity of two networks, extending previous approaches to network comparisons based on network topology^24^ and taking actual network response behaviour into account.

### Total centrality and value centrality

If 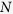 is a regulatory network, and 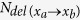 is identical to 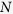 apart from the deletion of the connection from node 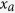 to node 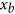, the total (control) centrality 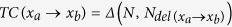 is a measure for the impact of null mutations of the connection. After deleting all connections originating in a node 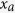 in the network 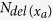, we obtain the centrality of the node 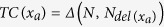 (Fig. 1g,h). For example, in the network from Fig. 1g, mutations of node B greatly affect network dynamics (TC_B_ = 0.48) by disrupting the central cycle B → C → D as well as disrupting the A → B → E pathway. Node E has a far smaller influence (TC_E_ = 0.10) since it is only involved in the B → E → F output pathway. This network motif applies e.g. for sub-networks modelling cell cycles.

The vulnerability to mutations of a network is intuitively defined as the mean of the total centralities 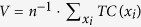. Conversely, 1-V is a measure of the robustness of the network.

The value centrality is an analogous concept quantifying the influence of the value of a node 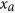 (which we assume to be the last node in the state vector for the sake of simplicity) on the convergence of the network. It is defined by


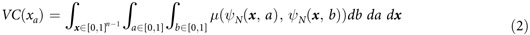


In a gene expression network, this formula corresponds to artificially altering the gene expression level of a given node 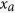 and examining whether the state of the network is affected. Gene expression omnibus gives numerous examples for such studies, e.g. gene expression changes after receptor changes.

The definition can be extended to connections 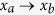 by splitting the input node 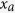 into an input node for the connection 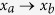, and another identical node for the rest of the network, using a node split function 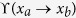 (Supplementary Methods 1.2).

In the network from Fig. 1g, the value of node B strongly influences the convergence of the network (VC_B_ = 0.21) since B can hold its state due to the self-amplifying loop (cf. for example the self-amplifying autocrine action of BDNF in axon development), and distribute it via the connections B → C and B → E. The value of node E on the other hand is overridden by the B → E connection (VC_E_ < 10^−10^).

The mean of the value centralities 
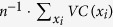
 is a measure of how well the network can be steered by the value of its nodes, i.e. its controllability.

Previous definitions of controllability^13^,^19^ considered subsets of the network nodes which could drive the network to any desired state, a concept that led to problems, for example because it grossly overestimated the number of essential driving nodes^21^. In contrast to these approaches, our definition considers the combined influence of the values of all network nodes, while abandoning the requirement to drive the network to any point in the state space.

It is reasonable to assume that biological networks can be controlled very well by the values of their nodes and are thus highly controllable according to our definition, while at the same time exhibiting robustness against node mutations. We will confirm both these predictions in all the biological networks surveyed below (Table 1).

The impact of mutations leading to permanent overexpression, under-expression or functional defects of a network node is best represented by its total centrality, whereas the value centrality captures the influence of the value of the node in the non-mutated regulatory system, covering physiological control by transcription factors, miRNA and chromatin regulators.

As a rough guide, in medium sized networks centralities above 10^−6^ represent strong influences, centralities between 10^−6^ and 10^−8^ are intermediate, and centralities below 10^−10^ are weak. Nodes with an intermediate influence cannot be ignored in their network influence, however, they will be dominated in their net effect by nodes with strong influence. If the value centralities of one or more nodes are considerably stronger than other value centralities in the network, they can be said to dominate the behaviour of the network, since the influence of the remaining nodes is negligible in comparison. In a biological context, these dominating nodes often represent essential master transcriptional regulators (see the T-helper network below).

### Dynamic centrality and sensitivities

Deleting a connection from the network intuitively removes two types of influence, the influence of the value of the node and a dynamic influence caused by the effect of the node on network dynamics. Dynamic centrality as introduced here captures and quantifies this dynamic network effect in biological networks.

We define the dynamic centrality 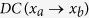 by trying to minimise the influence of the connection 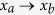 by segregating its input into a new node using the node split function and initialising the new node with an optimal neutral value 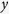:


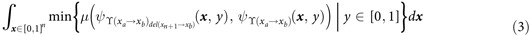


This definition is more difficult to grasp from a biological point of view. As an example, consider the regulation of blood glucose levels in humans by insulin and other hormones such as glucagon or incretins. For a given situation of this dynamic system, the definition tries to adjust the initial insulin concentration such that the system behaves as if insulin was not involved in the regulation of blood glucose at all. If this succeeds, only the initial insulin concentration affects the systems. If it fails, which is likely in this scenario, insulin affects the system beyond its initial concentration, for example because insulin relays regulatory stimuli from other parts of the regulatory system. This is what we define as dynamic centrality.

In the network from Fig. 1g, the influence of node B remains even after removing the influence of its initial value (DC_B_ = 0.16) due to its involvement in the B → C → D cycle and the A → B → E pathway. Node A, on the other hand, has no dynamic influence (DC_A_ < 10^−10^) since it is not part of any pathway.

In Supplementary Methods 1.3 we present an extension of the concept to nodes and show how the influence 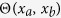 of a node 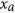 on another node 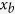 according to the three centralities 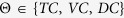 can be defined. This pairwise influence also leads to a definition of the sensitivity of a node 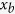 with respect to a certain centrality as 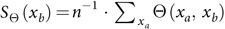.

The value sensitivity 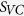 quantifies how sensitive the node is to variations in node values. It is high for nodes which are a good representation of the current network state.

## Results

### Control in random networks

We applied our definitions to random Erdős–Rényi (ER) networks, where connections are set with equal probability, as well as scale-free (SF) networks, where node degree distributions follow a power law. The number of network nodes with k connections to other nodes is in the latter proportional to k^−λ^ where λ is a constant close to 3 in the Barabási–Albert model we chose for this study for optimal comparison to previous studies. A large majority of naturally occurring networks are scale-free^29^. The generation of these random networks and of the biological networks summarised in Table 1 is described in detail in the supplementary material.

**Figure 2:**
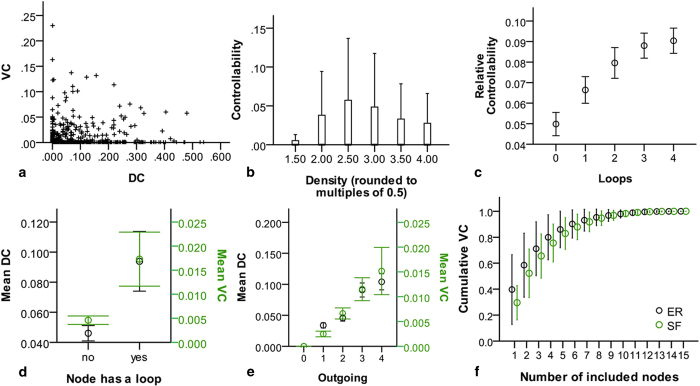
Controllability and centrality in random networks. (**a**) Weak correlation between dynamic (DC, x-axis) and value centrality (VC) in random networks (see Methods for network specifications). (**b**) High relative controllability (see text) is best achieved in sparsely connected networks with densities around 2.5 (upper end of the box: 75% percentile, outliers removed) (**c**) Self-amplifying loops increase the relative controllability (Error bars are  ±1SE). (**d**) Loops (right) increase dynamic (black, DC, left y-axis) and value centrality (VC, green, right y-axis) of nodes (Error bars are ±1SE). (**e**) Outgoing connections (x-axis) increase value and dynamic centrality (labels see d; error bars ±1SE). (**f**) The influence in random ER (black) or SF networks (green) resides in very few nodes (x-axis; y-axis shows cumulative value centrality of the most influential nodes, error bars are  ±1SD).

All calculations were performed in our simulation framework Jimena^30^ (Supplementary Methods 2). We find that the association between dynamic centrality and value centrality is significant (p < 0.001, Pearson’s correlation, n = 1324) but very weak (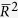 ≈ 0.015, linear regression, Fig. 2a). This intriguing observation shows that although both centralities measure the network influence of the node, they capture an almost independent aspect of its function. In a linear regression analysis, dynamic and value centrality account for 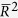 ≈ 0.98 of the variance in the total centrality, which confirms that they are its essential components. The centralities are thus tightly linked, and high controllability (mean value centrality) also necessitates a certain amount of vulnerability (mean total centrality). The same analysis in the biological networks below confirmed these results.

To assess the balance between a controllable and a robust network topology, we used the ratio between controllability and vulnerability which we call relative controllability. Considering this ratio helps to control for the distorting effect of functionally disconnected network nodes (appearing as artefacts in large, automatically generated networks), which artificially decrease the vulnerability but also decrease the controllability of the network. In random networks, the relative controllability of scale-free networks is maximal for networks where the density (connections to nodes ratio) is between 2.0 and 3.0 (Fig. 2b). According to the data, controllability has a pronounced maximum in this range, which suggests that a network is controlled better if sparsely connected.

Densities in this range are prevalent in biological networks, with a review^31^ reporting values between 1.4 and 2.75 and our own survey finding densities between 1.3 and 3.4. High relative controllabilities are biologically plausible since networks with low controllability would be too stiff to react to internal and external stimuli thus impairing their regulatory function, while high vulnerability would render the system more susceptible to deleterious mutations. It is therefore not surprising that the relative controllability of the biological networks below (Table 1) is high compared to random networks included in Fig. 2b.

Figure 2c shows that self-amplifying loops greatly increase the relative controllability (p < 0.001, Pearson’s correlation, n = 1199). This may contribute to their high conservation in evolutionary processes^32^. The influence is mainly exerted by increasing the mean value centrality (p = 0.006, Pearson’s correlation, n = 1199) and not by changing the mean total centrality (p = 0.24, Pearson’s correlation, n = 1199). This association can also be traced back to single nodes (Fig. 2d), where nodes with a self-amplifying loop have higher dynamic centrality (p = 0.002, independent samples t-test, n = 1324), value centrality (p < 0.001, independent samples t-test, n = 1324) and, interestingly, total centrality (p < 0.001, independent samples t-test, n = 1324).

We also calculated the Gini coefficient^33^,^34^ of the centrality distribution, a common measure for inequality, which is 0 if the influence is evenly distributed and approaches 1 for increasingly unequal distributions. In scale-free networks, we obtain a mean Gini coefficient of 0.71 for the total centralities and 0.68 for the value centralities, indicating very uneven distributions where the centrality is held by just a handful of nodes. For example, an average of 2 out of 15 nodes in random Erdős–Rényi and scale-free networks hold more than half of the total value centrality (Fig. 2f).

The properties of nodes which exert strong influence on the network are controversial, with Liu *et al.*^13^ asserting that driver nodes tend to avoid high degree nodes in linear networks. To shed light on this issue, we distinguish the number of connections originating in a node from the number of connections leading to it. Outgoing connections (Fig. 2e) substantially increase the value centrality (p < 0.001, Pearson’s correlation, n = 1324) and the dynamic centrality (p < 0.001, Pearson’s correlation, n = 1324), while incoming connections only increase the dynamic centrality (p < 0.001, Pearson’s correlation, n = 1324) but not the value centrality (p = 0.09, Pearson’s correlation, n = 1324). This is plausible since the value centrality only represents the influence of the value of the node itself.

Our results strongly suggest that on average high degree nodes influence network behaviour more than low degree nodes, not supporting Liu *et al.*^13^. This is plausible in biological systems, where the strength of a transcription factor increases with the number of target genes, as well as in artificial models, where the strength of a node increases with its appearances in the differential equations of other nodes.

The biological networks below further corroborated these results.

Further experimentation with random changes in network parameters such as relative influence strengths showed that driving nodes and other network functions are encoded in the network topology, and not in the specific choice of the parameters (see Supplementary Methods 1), providing robustness against modelling uncertainties.

### Controllability in biological networks

We examined controllability in prokaryotic and eukaryotic (animals, plant, fungi) regulatory networks (Table 1, see also Supplementary Tables 2–10 and Supplementary Figures 1–5).

In biological networks, control centrality is concentrated in an even smaller minority of nodes than in random networks (mean Gini(VC) = 0.78, SD = 0.18). The networks are sparsely connected and characterised by exceptionally high relative controllabilities compared to random networks (Fig. 2b).

For some networks, different perspectives on node functions are helpful. If self-amplifying loops are added to the input nodes, enabling them to hold a state, the reaction of the network to external stimuli is examined. Without these loops, the intrinsic behaviour without external stimuli is observed.

**Figure 3:**
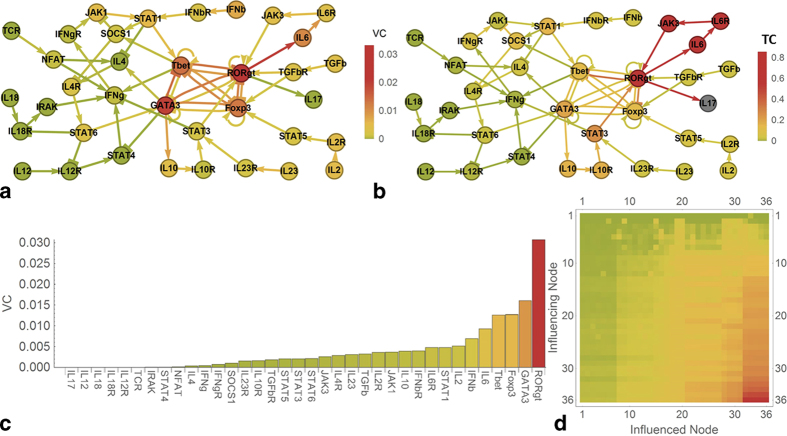
Intrinsic controllability in T-helper differentiation. (**a**) Value centrality in human T-helper cell differentiation^1^ (SQDS network) resides in the four central nodes Tbet, RORgt, GATA3 and Foxp3 (colour scheme: green-yellow-red from 0 to maximum controllability). (**b**) Total centrality on the IL17 node originates in the RORgt → IL6 → IL6R → JAK3 → RORgt cycle and to a lesser extent in the four central nodes. (**c**) Value centrality of the nodes in 3a. (**d**) Sensitivity matrix plot indicating the value influence of a node (y-axis) on each other node (x-axis). Nodes on both axes have been ordered by value centrality (y-axis, cf. Fig. 3c) and sensitivity (x-axis, Supplementary Table 2).

As an instructive and applied example, consider a network describing the differentiation of human T-helper cells^1^ without input loops. Figure 3a shows that the values of the genes Tbet, GATA3, RORgt and Foxp3, which according to experimental data^27^ and single null mutation analysis in silico^1^ are the master transcriptional regulators of the T helper subclasses Th1, Th2, Th17 and Treg, respectively. However, these master regulators are shown here to have also the strongest influence on convergence behaviour in their network (Fig. 3c) and therefore dominate network behaviour. In particular, high value centralities enable them to steer the network in the direction of the subclass they define, while a strong, well-connected central subnetwork between those nodes ensures that only one of the nodes is active (cf. the colour of the connections in Fig. 3a).

Blocking interleukin 6 (IL6), which is a strong inducer of Th17 cells in conjunction with transforming growth factor-beta^28^, strongly inhibits production of interleukin 17 (IL17) in T-helper cells according to experimental findings^26^. Furthermore, we exploited the ability of our concept to measure the influence of one network node on another. We plotted the total centrality of all network nodes and connections on IL17 (Fig. 3b). We found that the experimental result can be directly related to exceptionally high centralities in the RORgt → IL6 → IL6R → JAK3 →  RORgt cycle, with a TC influence of IL6 on IL17 of 0.85 (mean of the TC-influences of the nodes outside of this cycle on IL17: 0.05, SD: 0.07). Figure 3b also stresses that the influence from IL6 has to pass through the highly interconnected node RORgt (RAR-related orphan receptor gamma) to reach IL17.

Plotting all pairwise influences according to the value centrality (Fig. 3d) we show that the distribution of the sensitivities is very similar in all rows, indicating that the network is inter-connected well enough such that there is only one functional partition that reacts to stimuli from all network nodes.

**Figure 4:**
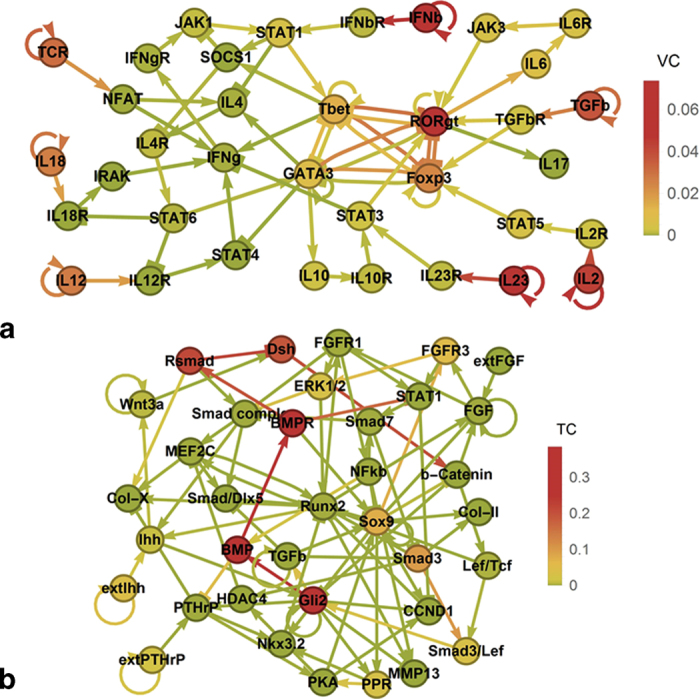
Extrinsic controllability in T-helper differentiation and chondrocytes. (**a**) When loops are added to the input nodes in T-helper differentiation, a balance between value centrality of external and internal stimuli is established. (**b**) Total centrality in chondrocyte proliferation and differentiation^2^ reveals a strong central subnetwork around the BMP pathway and the interference of Smad proteins with the Wnt pathway.

The situation changes visibly when loops are added to the input nodes (Fig. 4a) and parts of the influence of the four central nodes move to the input nodes. A significant amount of influence, however, remains with the central nodes, representing crosstalk between external and internal control in T-helper differentiation.

As another example with input loops, consider a network tested and confirmed to model the proliferation and differentation of chondrocytes in growth plates^2^ (Fig. 4b). Here we find several highly connected nodes and their main effectors exhibit high total centralities. Interestingly, these nodes, such as Sox9 (SRY box 9 , TC = 0.07, median 2.7·10^−4^), BMP (TC = 0.35) and Gli2 (GLI family zinc finger 2, TC = 0.32), have previously been experimentally identified as master regulators of chondrogenesis. They are all implicated in the development of chondrosarcomas^35^,^36^,^37^,^38^, thus corroborating the association between the total centrality and the impact of mutations or overexpression. The nodes calculated by our approach to have by far the highest value centrality in the network are the Indian hedgehog signal (extIhh, VC = 0.019, TC = 0.035) and parathyroid hormone-related protein (extPTHrP, VC = 0.010) which are known to be the two essential external stimuli of chondrogenesis^39^ (Supplementary Fig. 2).

Since therapeutic interventions by drugs or gene therapy exert a permanent influence on the target node, our new total centrality metric is well suited to identify potential target genes for intervention. Most of the aforementioned nodes with high total centralities (median TC = 2.7·10^−4^) turn out to be recently validated and important target nodes. In promising preclinical research, Sox9 gene therapy^40^ improves the healing of bone-tendon junctions and Indian hedgehog gene therapy^41^ induces chondrogenesis in mesenchymal stem cells. Bone morphogenetic proteins for bone regeneration have already been succesfully tested in clinical studies^42^.

Our approach elucidates furthermore interactions in important previously modelled and verified signalling pathways such as Wnt and BMP signalling^43^. The Wnt signal is transduced via the Dishevelled (Dsh) protein, which is modulated by the BMP pathways via receptor regulated Smad proteins (Rsmad). Calculating the total and the dynamic centralities in this system, we find a strong inhibiting influence in the connection from Rsmad to Dsh (Fig. 4b and Supplementary Fig. 2b). This heavily interferes with the Wnt signalling pathway, which is in keeping with experimental results showing that BMP-2 strongly inhibits Wnt induced β-Catenin production^43^.

The role of centralities is also instructive in a gene network involved in fibroblast differentiation to colon cells in the hindgut, experimentally studied and modelled recently^24^,^25^. In normal liver cells, which develop from the foregut, intestine specific markers such as homeobox transcriptional factor Cdx2 are not expressed in this network. In liver cells artificially induced from mouse embryonic fibroblast, however, an abnormal expression pattern of the colon network with a partially active hindgut programme is observed^24^,^25^.

The authors used their software CellNet to generate organ specific subnetworks from expression data and reverse engineering. They found 3 subnetworks specific for the colon identity of a cell. Within these networks, they also named the top 20 regulators causing this dysregulation according to a score that quantifies the dysregulation in the expression of target genes caused by mutations in transcriptional regulators^24^. For a detailed analysis of network convergence behaviour, we merged the 3 colon subnetworks into a large network with 1310 nodes and 16,742 connections, disregarding duplicate connections. We show that all 20 strong regulators exhibit high total centralities (mean 3.1·10^−3^, minimum: 6.2·10^−6^) and the master regulator Cdx2 shows an exceptionally high total centrality of 1.3·10^−2^. The value centrality of these nodes is negligible (mean 7.6·10^−19^), indicating that in this system the node influence is exerted via dynamic network effects (Supplementary Table 1 for additional data).

Jimena thus complements CellNet’s functions by revealing that stem cell node functions are exerted by dynamic network control in these transcriptional networks.

## Discussion

Biological network control is challenging. Various structural and mathematical definitions have been studied^19^,^44^,^45^,^46^,^47^, but results from *in silico* network models do not always agree with experimental data, often by overestimating the amount of nodes needed for effective control^13^,^21^,^22^.

Structural controllability^13^,^23^ has by its focus on network structure some inherent limitations, in spite of recently discussed^48^,^49^ extensions such as statistics on control node patterns^50^ (control profiles), describing the number of source, sink and external or internal dilation points from which structural controllability arises. We showed here how these shortcomings can be overcome by distinguishing directly steering nodes (value centrality) and network effects (dynamic centrality) with centrality metrics based on network convergence and therefore the fundamental static or dynamic equilibria governing most regulatory processes in biological systems^19^. The new metrics allow for novel insights into biological node functions, network pathways, controllability and robustness for instance regarding dominating nodes (cancer mutations, complex genetic diseases), system state behaviour over time (e.g. in differentiation, learning) as well as feedback loops (e.g. autocrine amplifiers) or cross-inhibition (e.g. TNFR1 versus TNFR2 networks). Their application in such systems biological investigations is only limited by the quality of currently available networks.

Our analysis shows that a large part of the value centrality is situated at the network borders, where external stimuli are fed into the system (e.g. via G-coupled transmembrane receptors as well as other important drug targets). With increasing integration of regulatory systems into more comprehensive, larger networks, there is a tendency that value centrality disappears from the former network borders and then replaced by more dynamic centrality and control.

The intriguingly weak association between value centrality and dynamic centrality we find in random Erdős–Rényi versus scale-free networks implies that these metrics arise from different topological factors, and we identify self-loops and the number of incoming connections (in-degree) to be contributing to the difference between these types of control.

We show specific biological examples for each type of control, extending earlier studies on T-cells^1^,^26^,^27^,^28^, complementing and adding insight to previous analyses on stem cell networks by CellNet^24^, as well as other networks (Table 1) and their analysis 2,39,43.

Since comprehensive databases on regulatory interactions such as the Human Transcriptional Regulation Interaction Database^51^ are containing a steeply increasing number of entries, we expect our new measures for control centralities to become an important tool to analyse these vast amounts of data. The results may shed further light on the evolutionary balance between the different types of network control revealed here, as well as further insights on robust network topologies, specific high-degree nodes dominating network behaviour, uneven distribution of centralities and other results of our analyses in random and biological networks.

## Additional Information

**How to cite this article**: Karl, S. and Dandekar, T. Convergence behaviour and Control in Non-Linear Biological Networks. *Sci. Rep.* **5**, 9746; doi: 10.1038/srep09746 (2015).

## Supplementary Material

Supplementary Information

## Figures and Tables

**Table 1:** **Controllability in eukaryotes and prokaryotes.**

**Manually created networks**	**Nodes**	**Interactions**	**Density**	**RC**	**Gini(VC)**	**Gini(DC)**	**Gini(TC)**
Human T-helper different.^1^ [with input loops]	36	62 [69]	1.7 [1.92]	0.113 [0.260]	0.655 [0.713]	0.713 [0.677]	0.642 [0.539]
Mammalian chondrocyte regulation^2^	36	91	2.5	0.016	0.951	0.826	0.811
A. thaliana inflorescence^10^	13	44	3.4	0.078	0.689	0.749	0.691
A. thaliana immune response^8^ (with input loops)	104	170	1.6	0.146	0.909	0.684	0.632
A. thaliana root stem cell niche^9^	13	27	2.1	0.230	0.820	0.836	0.735
S. pombe (fission yeast) cell cycle^6^	12	31	2.6	0.206	0.311	0.708	0.576
**Transcriptional factor networks**							
S. cerevisiae (budding yeast)^7^ [with input loops]	102	353 [371]	3.5 [3.6]	0.107 [0.281]	0.949 [0.842]	0.859 [0.829]	0.844 [0.728]
P. aeruginosa^12^ [with input loops]	87	112 [143]	1.3 [1.6]	0.174 [0.347]	0.908 [0.642]	0.881 [0.750]	0.832 [0.526]
E. coli^11^ [with input loops]	145	315 [341]	2.2 [2.4]	0.149 [0.357]	0.935 [0.825]	0.775 [0.691]	0.763 [0.664]

Density, relative controllability (RC) and Gini coefficient of the value centralities (VC), dynamic centralities (DC) and total centralities (TC) in biological networks. See Supplementary Methods 2 for more information on the networks.
