# Supplementary material for: Convergence behaviour and Control in Non-Linear Biological Networks
Source: Sci Rep. 2015 Jun 11;5:9746. doi: 10.1038/srep09746 (PMC4464179; doi:10.1038/srep09746)
Supplement: Supplementary Information [file srep09746-s1.pdf]

Convergence behaviour and Control in Non-Linear Biological Networks

Stefan Karl[1] & Thomas Dandekar[1,*]

Supplementary Information

As already detailed in our paper, application of our measures to other networks is most welcome, we provide a Java implementation of our concepts including a graphical user interface and the underlying code. Please visit our webpages for this (http://stefan-karl.de/jimena/, http://www.bioinfo.biozentrum.uni-wuerzburg.de/computing/jimena-c). We provide also a starting tutorial (click "Centralities tutorial") with simple application examples there according to the results presented in the paper.

Supplementary Methods

1 Mathematical description of the centralities

1.1 Convergence and network comparisons

A time-continuous regulatory network is defined by a vector of nodes, and corresponding differential equations $\dot{x}_i(t) = f_i(\boldsymbol{x}(t))$ where $\boldsymbol{x}(t) = (x_1(t),\ldots,x_n(t))$ is the state vector of the network at time index t. Each function f_i potentially defines multiple *connections*, i.e. influences of node values $x_{j_1}, x_{j_2}\ldots$ on the node x_i which are usually depicted as arrows in network graphs. In the usual modelling frameworks, connections can be removed from f_i, for example by ignoring the activating or inhibiting influence when constructing the differential equations of the SQDS (standardised qualitative dynamical systems) method (reference in the paper). This is equivalent to deleting an arrow in a network graph.

To define our control centralities, it is necessary to quantify the difference in behaviour of two related regulatory networks. Comparing time series data of responses to stimuli is not justified due to

[1] Department of Bioinformatics, University of Würzburg, Am Hubland, 97074 Würzburg, Germany
* Corresponding author

the general lack of kinetic data. We therefore base our definitions on the *convergence behaviour* ψ of the network, which exhibits a robustness against kinetic modelling uncertainties.
For a formal definition, consider two networks N_1 and N_2 with identical vectors of nodes. If $\boldsymbol{x} \in [0,1]^n$ is a state vector of the network N (i.e. a vector containing the values of all network nodes), we define $\psi_N(\boldsymbol{x})$ as the state vector the network N converges to when simulated for time indices $t \to \infty$ from the initial state vector $\boldsymbol{x}$. The codomain of ψ_N are the stable states of the network since the expression is undefined if the network state does not converge. For two (state) vectors $\boldsymbol{x}, \boldsymbol{y} \in [0,1]^n$ the mean squared difference is $\mu_S(\boldsymbol{x}, \boldsymbol{y}) = |S|^{-1} \cdot \sum_{i \in S} (x_i - y_i)^2$ where $S \subseteq \{1, \ldots, n\}$ represents a subset of the network nodes which are considered *significant*, for example biologically significant effector nodes. If no significant nodes are given, all nodes are considered significant.
We then define the convergence difference of two networks with the same nodes as

$$\Delta_S(N_1, N_2) = \int_{\boldsymbol{x} \in [0,1]^n} \mu_S\left(\psi_{N_1}(\boldsymbol{x}), \psi_{N_2}(\boldsymbol{x})\right) d\boldsymbol{x}$$

It is implicitly assumed, that the integration domain in all integrals is limited to points where the integrand exists, and that the results of the integrations in these cases are scaled such that they represent the mean value of the integrand. In practice, we approximate the integrals numerically by random sampling.
Note that the existence of this and other integrals in the article cannot be guaranteed in a strict mathematical sense, the calculations do, however, converge in practice.

1.2 Total centrality and value centrality

Using the convergence difference Δ_S, we quantify the influence of network manipulations on the behaviour of a network. If N is a regulatory network, and $N_{del(x_a \to x_b)}$ is identical to N apart from the deletion of the connection from a node x_a to another node x_b, the *total (control) centrality* (TC) $TC_{N,S}(x_a \to x_b) = \Delta_S\left(N, N_{del(x_a \to x_b)}\right)$ is a measure of the influence of the connection on network behaviour. After deleting all connections originating in a node x_a in the network $N_{del(x_a)}$ we obtain the centrality of the node $TC_{N,S}(x_a) = \Delta_S\left(N, N_{del(x_a)}\right)$, which is a measure of the impact of null mutations of the node.
The *vulnerability* V of a network, a measure of the susceptibility of the network to mutations, may be intuitively defined as the mean of the total centralities $n^{-1} \cdot \sum_{x_a} TC_N(x_a)$. Conversely, $1 - V$ is a measure of the *robustness* of the network.

An analogous concept which quantifies the influence of the value of a node x_a, which we assume to be the last node in the state vector for the sake of simplicity, is the *value control centrality* (VC) defined by

$$VC_{N,S}(x_a)=\int_{\boldsymbol{x}\in[0,1]^{n-1}}\int_{a\in[0,1]}\int_{b\in[0,1]}\mu_S(\psi_N(\boldsymbol{x},a),\psi_N(\boldsymbol{x},b))\,db\,da\,d\boldsymbol{x}$$

In short, we compare the convergence of the network starting from random pairs of initial vectors which differ in only one component.

To define $VC_{N,S}$ for single connections, we need to split the node the connection originates in. For a given connection $x_a \to x_b$ in a network N with nodes $x_1,...,x_n$, we construct the network $\Upsilon_N(x_a \to x_b)$ with nodes $x_1,...,x_n,x_{n+1}$ and functions $\tilde{f}_i$ where $\forall i \notin \{n+1,b\}: \tilde{f}_i(\boldsymbol{x})=f_i(\boldsymbol{x})$, $\tilde{f}_{n+1}(\boldsymbol{x})$ is equal to $f_a(\boldsymbol{x})$ except for all occurrences x_a which are replaced by x_{n+1} and $\tilde{f}_b(\boldsymbol{x})$ is equal to $f_b(\boldsymbol{x})$ except for all occurrences of x_a which are replaced by x_{n+1}. The value centrality is then given by

$$VC_{N,S}(x_a \to x_b)=\int_{\boldsymbol{x}\in[0,1]^{n}}\int_{a\in[0,1]}\int_{b\in[0,1]}\mu_S\left(\psi_{\Upsilon_N(x_a\to x_b)}(\boldsymbol{x},a),\psi_{\Upsilon_N(x_a\to x_b)}(\boldsymbol{x},b)\right)db\,da\,d\boldsymbol{x}$$

In other words, we split the node x_a into two identical nodes x_a and x_{n+1}, where x_{n+1} takes the place of x_a for the connection $x_a \to x_b$ and then quantify the influence of the value of x_{n+1} on the convergence behaviour.

The mean of the value centralities $n^{-1}\cdot\sum_{x_a} VC_N(x_a)$ is a measure of how well the network can be steered by the value of its nodes, i.e. its *controllability*.

The impact of mutations leading to permanent overexpression, underexpression or functional defects of a network node such as a protein is best given by its total centrality, since the value centrality only captures the influence of the initial value of the node, whereas the total centrality represents a permanent influence.

1.3 Dynamic centrality and sensitivities

Deleting a connection from the network intuitively removes two types of influence from the network, the influence of the initial value of the node and a dynamic influence caused by the effect of the node on network dynamics. The latter concept is especially difficult to define since in some network models such as SQDS, a connection $x_a \to x_b$ exerts a constant influence even beyond the value of its source node, i.e. there is no possible value $y \in [0,1]$ of the source node which would mimic the effect of completely deleting the input from its function f. If specific values for the other input nodes of

f and the target node are assumed, such a value can however usually be found for the specific situation. It is therefore possible to define the *dynamic centrality* $DC_{N,S}(x_a \to x_b)$ of a connection $x_a \to x_b$ by constructing $\Upsilon_N(x_a \to x_b)$ and determining

$$\int_{\boldsymbol{x}\in[0,1]^n} \min\left\{\mu_S\left(\psi_{\Upsilon_N(x_a\to x_b)_{del(x_{n+1}\to x_b)}}(\boldsymbol{x},y),\psi_{\Upsilon_N(x_a\to x_b)}(\boldsymbol{x},y)\right)\middle| y\in[0,1]\right\}d\boldsymbol{x}$$

In other words, we compare the network convergence behaviour between the network where the connection has been deleted and the network where it has been initialised with the optimal neutral initial value y for the connection $x_a \to x_b$. If the difference is high, the node can be assumed to influence the network independently of its initial value, for example by relaying the excitation of other nodes.

An analogous measure $DC_{N,S}(x_a)$ for nodes is defined by splitting the node x_a with k outgoing connections into k nodes $x_{a,1},...,x_{a,k}$ by repeated application of $\Upsilon_N(x_a \to ...)$ and searching for optimal neutral initial values $y_1,...,y_k \in [0,1]$ for those connections.

By restricting the calculation of the network difference to a node x_c, we get the total centrality, value centrality and dynamic centrality of a connection $x_a \to x_b$ of a node x_a on the node x_c:

$$\Theta_N(x_a \to x_b, x_c) = \Theta_{N,\{x_c\}}(x_a \to x_b)$$
$$\Theta_N(x_a, x_c) = \Theta_{N,\{x_c\}}(x_a)$$
$$\text{where } \Theta \in \{TC, VC, DC\}$$

We can also define the sensitivity of a node x_c with respect to a certain centrality as $S_{N,\Theta}(x_c)$ $= |N|^{-1} \cdot \sum_{x_a} \Theta_N(x_a, x_c)$ where $\Theta \in \{TC, VC, DC\}$ and $|N|$ is the number of nodes in the network N. The value sensitivity $S_{N,VC}$ quantifies how much the value of a node varies if manipulations of the initial vector change the convergence behaviour, and is therefore high for nodes whose values separate different stable states and are a good representation of the current network status. $S_{N,TC}$ on the other hand is a measure of the susceptibility of the node to mutations in the network.

Experimentation with numerical parameters underlying the models such as thresholds showed that the centralities and derived networks metrics react benignly to variations in these parameters, with the relative change in the metrics being proportional to the relative change in the parameters. This leads to a robust analysis of nodes functions, considering that strong nodes have centralities in the order of 10^{-1}, whereas weak nodes exhibit values smaller than 10^{-8}, such that relative changes of ±50 % are negligible.

2 Calculation details

Number of samples for the approximations

The survey of random and biological networks was conducted in our regulatory network analysis framework Jimena[1], which is available at http://stefan-karl.de/jimena/jimenapreview.php
We implicitly assume that the integration domain in all integrals is limited to points where the integrand exists, and that the results of the integrations in these cases are scaled such that they represent the mean of the integrand.
In Jimena, the integrals and the minimum in the calculation of the dynamic centrality were approximated numerically by random sampling with different numbers of samples:

- Mouse colon subnet: TC/VC: 100
- Human T-helper differentiation: TC/VC: 1000, DC (integral): 100, DC (minimum): 50
- Mammalian chondrocyte regulation: TC/VC: 1000, DC (integral): 80, DC (minimum): 50
- *A. thaliana* inflorescence: TC/VC: 1000, DC (integral): 100, DC (minimum): 50
- A. *thaliana* immune response: TC/VC: 100, DC (integral): 20, DC (minimum): 20
- *A. thaliana* root stem cell niche: TC/VC: 1000, DC (integral): 150, DC (minimum): 80
- *S. pombe* (fission yeast) cell cycle: TC/VC: 100, DC (integral): 100, DC (minimum): 50
- *S. cerevisiae* (budding yeast): TC/VC: 50, DC (integral): 10, DC (minimum): 10
- *P. aeruginosa*: TC/VC: 50, DC (integral): 10, DC (minimum): 10
- *E. coli*: TC/VC: 50, DC (integral): 10, DC (minimum): 10

In large networks only a small number of simulations per node are necessary for accurate and reproducible results due to the large state vectors, and more simulations are computationally prohibitive.

Specifications of the random networks in Figure 2

a Scale-free and Erdős-Rényi, 15 nodes, 25 connections. **b** SF networks, 20 or 40 nodes, 30-80 or 60-160 connection, respectively. **c** Scale-free, 20 nodes, 50 connections. **d** Scale-free and Erdős-Rényi, 15 nodes, 25 connections. **e** Erdős-Rényi and scale-free, 15 nodes, 25 connections. **f** Scale-free, 15 nodes, 25 connections, 0 to 4 loops

The Gini coefficient

The Gini coefficient[2,3] of the centralities was calculated according to

$$Gini(x_1,...,x_n) = \frac{\sum_{i=1}^{n}\sum_{j=1}^{n}|x_i - x_j|}{2n^2\sum_{i=1}^{n}x_i} \text{ for values } x_1,...,x_n$$

which is 0 if all nodes react equally strongly to influences from other nodes, and approaches 1 for increasingly unequal distributions.

Generation of the random networks

Erdős-Rényi networks were created by randomly distributing the desired number of edges over the nodes and discarding the result if the network is not fully connected. Scale-free networks were grown to the desired size by preferential attachment according to the Barabási-Albert model: Starting from a seed of 2 nodes connected by either an activating or an inhibiting connection, new nodes were iteratively added to the existing network. The number of connections each of these new nodes has to the existing network was chosen in accordance with the desired number of connections in the generated network. The type of the interactions was chosen randomly between inhibiting (50%) and activating (50%) influences. If specific network parameters such as the number of input nodes were desired, random networks were fulfilling these criteria were chosen randomly.

Generation of the genome-wide networks

For the construction of the transcription factor networks for E. coli, P. aeruginosa and S. cerevisiae, we retrieved the regulatory influences between transcription factors evidenced by expression data from the databases in the citations. From this interaction data, we determined the names of the genes which appeared as the source of a regulation at least once. These genes were taken as the nodes of the regulatory network. All interactions between these nodes were then extracted from the source data and modelled in the network as activating or inhibiting influences with a relative strength of 1 (see also Supplementary Information section II). These networks and the mouse colon subnetwork were simulated using the SQDS template with standard parameters.
All remaining networks were drawn manually in the yEd Graph Editor version 3.14 and imported into Jimena.

Numerical parameters of the simulations

Unless otherwise specified, network parameters were set to a decay constant of 1 in the SQDS and the Odefy models, parameters n = 2 and k = 2 for Odefy's Hill functions, and a steepness parameter of h = 10 in the SQDS model.

Supplementary Tables: Additional information on the biological networks

All networks were simulated using the SQDS template with standard parameters to ensure comparability, except for the A. thaliana inflorescence network which has been modelled using Boolean expressions and simulated using the Odefy normalized HillCube method.
References to the sources of the networks are given in the main text.

Supplementary Table 1: Mouse colon subnetwork

The networks colon_subnet_1 (10,361 connections), colon_subnet_2 (4,711 connections) and colon_subnet_3 (3,865 connections) from the CellNet website (http://cellnet.hms.harvard.edu/grn/mouse, as of 14.10.2014) were merged into one large subnetwork with 1310 nodes and 16,742 connections. Duplicate connections were discarded, and loops added to all input nodes.
The network was simulated using the SQDS model with standard parameters.

Node	TC	VC
cdx2	1.32E-02	6.57E-20
klf5	8.46E-04	2.76E-21
hoxa9	7.93E-06	1.85E-21
hoxb13	9.85E-03	7.39E-21
vdr	4.69E-03	1.08E-20
hoxd10	7.71E-04	1.41E-21
foxd2	8.21E-04	3.00E-18
hoxa10	1.04E-02	4.27E-19
satb2	1.02E-03	4.52E-21
hoxa11	2.33E-05	7.15E-21
hoxd9	4.88E-03	1.77E-20
gfi1	8.30E-03	1.77E-18
klf4	6.20E-06	1.12E-21
hoxb9	3.25E-05	2.59E-21
atoh1	1.54E-03	8.67E-18
ovol2	1.28E-03	1.03E-18
hoxd13	2.66E-03	7.13E-20
pitx1	1.67E-03	6.65E-20
grhl2	7.83E-04	2.93E-21
ppard	1.27E-04	6.70E-21

Supplementary Table 2: Human T-helper differentiation

A manually modelled network describing the differentiation of human T-helper cells to the effector types Th1, Th2, Th17 and Treg.
The high TC and DC values of some input nodes such as IL18 represent mathematical characteristics of the SQDS mathematical template where a node influences the differential equations not only with its value.

In the source publication, the network was simulated with an arbitrary SQDS steepness value of 50 for all nodes, improving the reaction of the Foxp3 node (VC sensitivity). We simulated the network with the standard SQDS steepness of 10 to ensure comparability with the other networks.

Node	TC	VC	DC	TC-Sens.	VC-Sens.	Outgoing	Incoming	Loop
Foxp3	3.1E-02	1.3E-02	2.7E-03	3.1E-04	1.4E-16	4	6	yes
GATA3	2.7E-02	2.0E-02	3.0E-16	1.3E-02	1.2E-03	7	5	yes
IFNb	6.6E-03	7.1E-03	1.2E-16	1.2E-19	3.5E-20	1	0	no
IFNbR	9.5E-03	2.2E-03	6.7E-03	3.6E-18	1.0E-18	1	1	no
IFNg	1.3E-03	6.4E-04	5.2E-04	8.8E-02	3.1E-03	1	5	no
IFNgR	8.0E-02	5.1E-04	6.8E-02	1.1E-01	5.7E-03	1	1	no
IL10	2.1E-02	4.1E-03	1.4E-02	1.3E-02	1.2E-03	1	1	no
IL10R	3.1E-02	7.2E-04	2.4E-02	1.3E-02	1.2E-03	1	1	no
IL12	1.4E-01	5.3E-19	1.3E-01	1.3E-19	4.6E-20	1	0	no
IL12R	1.0E-01	6.8E-19	1.1E-01	2.7E-02	6.5E-19	1	2	no
IL17	0.0E+00	4.0E-21	0.0E+00	1.4E-01	2.6E-02	0	1	no
IL18	1.4E-01	1.3E-18	1.4E-01	1.4E-19	4.2E-20	1	0	no
IL18R	6.4E-04	1.4E-18	5.9E-18	2.7E-02	5.6E-19	1	2	no
IL2	3.7E-03	4.8E-03	1.6E-16	1.2E-19	3.3E-20	1	0	no
IL23	3.9E-03	2.8E-03	1.7E-17	1.4E-19	4.3E-20	1	0	no
IL23R	3.9E-03	1.5E-03	2.7E-03	4.3E-18	1.3E-18	1	1	no
IL2R	6.7E-03	4.5E-03	2.2E-03	3.6E-18	9.6E-19	1	1	no
IL4	8.1E-02	2.4E-17	8.1E-02	1.1E-02	1.1E-03	1	3	no
IL4R	1.8E-03	2.2E-03	8.6E-17	3.5E-02	1.2E-03	1	2	no
IL6	1.2E-01	1.1E-02	9.1E-02	1.4E-01	2.6E-02	1	1	no
IL6R	1.2E-01	3.3E-03	1.1E-01	1.4E-01	2.6E-02	1	1	no
IRAK	3.8E-06	3.1E-19	1.6E-06	2.7E-02	4.1E-18	1	1	no
JAK1	6.7E-03	4.6E-03	8.3E-10	3.0E-03	1.4E-07	1	2	no
JAK3	1.2E-01	4.2E-03	1.2E-01	1.4E-01	2.6E-02	1	1	no
NFAT	1.3E-03	8.1E-04	3.2E-03	4.3E-18	1.3E-18	2	1	no
RORgt	1.5E-01	3.3E-02	9.1E-02	1.3E-01	2.4E-02	6	7	yes
SOCS1	2.7E-02	1.2E-03	2.9E-02	9.1E-02	4.8E-03	2	2	no
STAT1	3.6E-02	5.5E-03	3.3E-02	2.6E-03	1.6E-09	3	2	no
STAT3	4.1E-02	1.7E-03	2.5E-02	1.1E-02	1.1E-03	2	2	no
STAT4	5.4E-06	7.9E-20	3.2E-06	5.3E-02	3.5E-18	1	2	no
STAT5	8.4E-03	1.9E-03	1.6E-03	4.6E-17	1.2E-17	1	1	no
STAT6	4.8E-03	2.8E-03	4.6E-03	3.5E-02	1.2E-03	3	1	no
Tbet	2.6E-02	1.4E-02	3.2E-03	9.1E-02	4.8E-03	6	5	yes
TCR	1.7E-17	8.3E-18	5.9E-18	1.3E-19	4.2E-20	1	0	no
TGFb	4.8E-03	3.6E-03	5.9E-17	1.3E-19	4.1E-20	1	0	no
TGFbR	4.8E-03	2.7E-03	3.2E-03	4.1E-18	1.2E-18	2	1	no

Supplementary Table 3: Chondrocyte regulation

A manually modelled network describing chondrocyte proliferation and differentiation in growth plates.

Node name	TC	VC	DC	TC-Sens.	VC-Sens.	Outgoing	Incoming	Loop
b-Catenin	2.8E-16	2.1E-16	1.6E-16	4.0E-02	1.3E-11	3	2	no
BMP	3.5E-01	1.1E-16	3.3E-01	3.5E-02	1.8E-07	2	2	no
BMPR	3.8E-01	1.6E-16	4.0E-01	6.3E-02	4.9E-10	3	1	no
CCND1	7.2E-11	1.1E-16	7.2E-11	1.1E-01	9.2E-11	1	4	no
Col-II	0.0E+00	9.8E-22	0.0E+00	4.7E-02	6.7E-10	0	2	no
Col-X	0.0E+00	6.2E-22	0.0E+00	6.6E-02	2.8E-08	0	4	no
Dsh	1.9E-01	1.0E-16	1.9E-01	1.1E-01	1.1E-14	1	2	no
ERK1/2	2.7E-02	1.1E-16	2.6E-02	4.6E-02	8.2E-11	2	2	no
extFGF	1.7E-16	1.3E-16	5.4E-17	5.8E-20	1.4E-20	1	0	no
extIhh	3.5E-02	1.9E-02	4.8E-07	2.0E-02	1.1E-02	2	1	yes
extPTHrP	2.1E-02	1.0E-02	8.8E-12	2.0E-02	1.0E-02	2	1	yes
FGF	1.5E-04	3.6E-16	1.5E-04	4.5E-02	3.2E-14	3	5	yes
FGFR1	2.5E-04	5.7E-16	2.5E-04	4.6E-02	3.7E-12	3	3	no
FGFR3	5.0E-02	9.7E-17	5.0E-02	2.4E-02	6.7E-10	2	2	no

Gli2	3.2E-01	1.7E-16	3.3E-01	6.4E-02	2.3E-06	7	4	yes
HDAC4	1.2E-05	1.2E-17	1.2E-05	4.1E-02	5.8E-08	1	2	no
Lef/Tcf	2.0E-03	8.2E-17	1.4E-04	4.0E-02	2.6E-13	2	1	no
lhh	1.1E-02	1.4E-04	4.7E-03	3.7E-02	8.2E-03	2	3	no
MEF2C	2.1E-04	3.6E-17	2.1E-04	3.5E-02	3.4E-13	3	3	no
MMP13	0.0E+00	1.1E-21	0.0E+00	4.6E-02	3.8E-12	0	3	no
NFkb	1.4E-04	4.0E-16	1.4E-04	4.7E-02	8.2E-14	4	1	no
Nkx3.2	7.1E-11	1.0E-16	7.1E-11	4.0E-02	1.7E-08	1	3	no
PKA	2.6E-08	1.2E-16	2.6E-08	6.0E-02	5.2E-07	3	2	no
PPR	2.4E-02	9.5E-17	2.4E-02	4.7E-02	9.7E-10	2	2	no
PTHrP	1.4E-04	9.9E-17	1.4E-04	4.9E-02	4.8E-05	3	3	no
Rsmad	2.2E-01	1.2E-16	2.4E-01	8.8E-02	5.7E-13	3	1	no
Runx2	3.5E-04	4.3E-16	3.1E-04	4.0E-02	1.3E-10	7	7	no
Smad	1.4E-04	3.3E-16	4.6E-05	6.9E-02	1.7E-14	4	3	no
Smad/Dlx5	3.6E-13	2.6E-17	3.6E-13	4.3E-03	1.0E-14	1	3	no
Smad3	9.5E-02	9.1E-05	9.7E-02	1.1E-01	3.6E-13	4	2	no
Smad3/Lef	2.9E-02	3.5E-17	3.9E-02	8.2E-02	2.8E-14	1	2	no
Smad7	2.9E-04	9.0E-17	2.8E-04	9.8E-02	1.2E-11	2	2	no
Sox9	7.5E-02	3.5E-16	7.4E-02	4.6E-02	5.8E-09	8	6	yes
STAT1	4.9E-12	1.8E-16	4.9E-12	7.6E-02	8.2E-11	2	3	no
TGFb	1.0E-05	1.2E-16	1.0E-05	1.2E-02	3.1E-10	4	2	yes
Wnt3a	5.4E-03	4.5E-05	6.1E-03	1.3E-02	5.8E-04	2	2	yes

Supplementary Table 4: *A. thaliana* inflorescence

A manually modelled network describing the differentiation of primordial cells in Arabidopsis thaliana during early flower development.

Node name	TC	VC	DC	TC-Sens.	VC-Sens.	Outgoing	Incoming	Loop
AG	1.7E-01	4.0E-03	1.7E-01	5.5E-02	3.8E-03	5	8	yes
AP1	2.5E-02	2.3E-03	1.7E-02	8.4E-02	3.4E-03	5	6	no
AP2	1.5E-03	1.5E-03	1.8E-16	8.7E-02	4.6E-04	1	1	no
AP3	1.2E-02	3.5E-03	6.9E-03	8.4E-02	3.4E-02	3	7	yes
EMF1	1.6E-01	5.5E-03	2.0E-01	4.9E-02	4.6E-04	4	1	no
FT	1.5E-03	6.9E-17	2.3E-03	1.2E-01	4.6E-04	1	1	no
FUL	0.0E+00	3.0E-20	0.0E+00	9.4E-02	3.8E-03	0	2	no
LFY1	1.5E-01	6.3E-03	1.4E-01	4.9E-02	4.6E-04	7	2	no
PI	4.8E-03	3.4E-03	7.7E-04	6.6E-02	1.2E-03	3	6	yes
SEP	3.1E-03	1.7E-03	1.5E-03	1.3E-01	4.6E-04	4	1	no
TFL1	4.0E-01	1.5E-03	4.6E-01	4.9E-02	4.6E-04	5	3	no
UFO	7.0E-02	4.7E-02	3.8E-03	5.8E-02	2.8E-02	2	1	yes
WUS	1.4E-03	6.2E-04	2.1E-16	8.1E-02	4.6E-04	2	3	yes

Supplementary Table 5: *A. thaliana* immune response

A manually modelled network describing the immune response of *Arabidopsis thaliana* against the gram-negative bacterium *Pseudomonas syringae* pv tomato DC3000. Input loops have been added to better represent the external stimuli.

Node name	TC	VC	DC	TC-Sens.	VC-Sens.	Outgoing	Incoming	Loop
A ARRs	2.8E-03	1.7E-20	2.1E-03	3.2E-02	1.0E-03	1	1	no
AAO	1.7E-04	1.8E-18	3.4E-03	5.0E-02	3.4E-03	1	1	no
ABA	1.5E-02	8.7E-04	1.8E-02	9.9E-03	3.7E-03	4	4	no
ACS	3.4E-02	2.4E-19	5.7E-02	4.1E-02	2.0E-03	1	1	no
AFB1	2.9E-03	1.5E-21	7.5E-03	9.5E-03	2.3E-03	1	1	no
AGT	3.2E-02	1.8E-02	1.3E-21	7.7E-03	3.3E-03	2	1	yes
AHKs	1.9E-05	2.5E-17	2.8E-05	5.0E-03	7.4E-07	1	1	no
AHP	2.6E-02	6.8E-18	1.7E-02	2.3E-02	9.1E-04	1	2	no
AOC	9.5E-03	5.0E-23	9.2E-03	2.8E-02	5.6E-03	1	1	no
AOS	1.8E-02	1.7E-21	2.1E-02	1.9E-02	5.0E-03	1	2	no

ARF1	8.5E-04	1.2E-21	9.0E-04	1.5E-02	3.9E-03	2	2	no
ASD	7.5E-02	5.2E-17	6.8E-02	5.0E-03	2.0E-03	1	1	no
ATK	7.9E-02	2.5E-02	1.1E-16	5.0E-03	2.0E-03	2	1	yes
Aux	3.1E-02	4.1E-17	7.0E-02	6.9E-03	2.3E-03	5	3	no
Aux/IAA	1.8E-06	2.5E-25	2.0E-06	1.5E-02	2.0E-03	1	1	no
Avr PtoB	4.5E-02	8.9E-18	3.6E-02	7.9E-03	2.7E-03	4	1	no
Avr Rpm1	2.6E-21	7.9E-21	1.3E-27	7.9E-03	2.7E-03	1	1	no
Avr RPT2	7.7E-03	2.7E-20	1.6E-05	7.9E-03	2.7E-03	2	1	no
B ARRs	1.5E-02	1.2E-17	1.5E-02	3.0E-02	1.0E-03	4	1	no
BRH	9.9E-03	1.4E-21	1.4E-02	4.0E-02	3.4E-03	1	1	no
Callo.	8.6E-05	1.1E-24	1.1E-04	9.6E-03	5.5E-23	1	1	no
CED	2.7E-02	1.3E-21	2.3E-02	2.8E-02	3.4E-03	1	1	no
CK	1.2E-06	5.0E-17	1.2E-06	5.1E-03	9.0E-06	1	3	no
CKX	1.9E-05	2.7E-17	1.7E-05	3.2E-02	1.0E-03	1	1	no
C-LRR	7.5E-03	2.5E-23	8.4E-03	7.9E-03	2.7E-03	1	3	no
COR	2.6E-04	8.4E-21	1.8E-03	7.9E-03	2.7E-03	1	1	no
CTH	4.1E-05	1.3E-17	3.1E-05	1.4E-02	1.7E-03	1	1	no
CTL	5.4E-02	5.0E-18	7.8E-02	2.5E-02	2.0E-03	1	1	no
DELLA	4.2E-03	9.2E-18	1.3E-04	4.2E-02	5.1E-03	5	3	no
DPS	2.2E-02	1.3E-02	7.9E-20	7.2E-03	4.2E-03	2	1	yes
EDS	6.9E-02	3.6E-02	3.6E-03	7.3E-03	3.7E-03	2	1	yes
EF	2.4E-02	2.8E-20	2.4E-02	7.9E-03	2.7E-03	1	1	no
EFR	1.6E-02	8.8E-03	1.1E-22	7.3E-03	4.7E-03	2	1	yes
EIN2	1.2E-02	2.3E-21	1.4E-02	2.6E-02	1.0E-03	3	1	no
EIN3	2.2E-03	3.7E-23	2.3E-03	3.0E-02	1.1E-03	1	1	no
EKO	4.4E-02	4.5E-19	4.4E-02	1.4E-02	3.7E-03	1	1	no
EKS	5.3E-02	7.9E-04	4.2E-02	7.3E-03	3.7E-03	1	1	no
ERF1	4.6E-03	3.9E-26	8.1E-03	3.9E-02	3.7E-03	1	2	no
ET	2.2E-02	5.5E-17	2.7E-02	2.1E-02	9.2E-04	2	2	no
ETR/CTR1	3.4E-03	1.4E-17	9.5E-03	2.6E-02	1.0E-03	2	1	no
EUO	4.0E-02	1.1E-03	3.7E-02	2.2E-02	3.7E-03	1	1	no
Flag	4.7E-02	3.0E-17	4.3E-02	7.9E-03	2.7E-03	1	1	no
FLS2/BAK1	3.0E-03	3.1E-03	3.8E-03	9.6E-03	2.6E-23	5	2	no
GA	3.6E-02	3.7E-03	2.6E-02	5.0E-02	6.2E-03	2	3	no
GID1-rec	3.0E-02	4.1E-03	2.4E-02	5.5E-02	6.6E-03	2	1	no
GRX480	1.3E-03	6.0E-23	2.4E-07	3.3E-02	2.1E-03	2	1	no
GTO/GHO	1.2E-02	8.3E-18	2.0E-02	2.9E-02	3.7E-03	1	1	no
HOP	2.4E-02	2.7E-21	2.1E-02	7.9E-03	2.7E-03	2	1	no
HSD	4.6E-02	5.1E-17	6.1E-02	9.9E-03	2.0E-03	1	1	no
HSK	6.0E-02	1.2E-17	5.2E-02	1.5E-02	2.0E-03	1	1	no
IAA Syn.	7.0E-02	2.4E-02	1.0E-03	5.4E-03	2.6E-03	3	1	yes
IAD	1.9E-02	3.3E-18	8.4E-03	6.6E-03	3.1E-03	1	1	no
IAN/IAO/ICO	2.7E-03	8.5E-19	2.0E-02	1.4E-02	3.1E-03	1	1	no
ICS/PAL	2.3E-05	8.0E-22	4.2E-08	1.5E-02	4.2E-03	1	1	no
IDI	6.7E-02	2.9E-17	7.7E-02	4.7E-03	3.4E-03	2	1	no
IPT	3.1E-03	5.2E-19	3.5E-03	1.1E-02	1.7E-03	1	2	no
JA	1.8E-03	5.8E-22	8.8E-06	3.4E-02	4.7E-03	1	3	no
JAZ	2.1E-03	3.5E-24	6.5E-04	2.3E-02	3.3E-03	2	2	no
LBC	1.5E-02	2.2E-22	1.6E-02	3.5E-02	3.4E-03	1	1	no
LOX	6.0E-03	4.2E-22	7.5E-03	7.3E-03	3.6E-03	1	1	no
LOX2	0.0E+00	3.6E-26	0.0E+00	2.1E-02	3.3E-03	0	2	no
MAPK1	2.5E-05	3.8E-23	1.6E-05	2.2E-02	2.9E-03	1	1	no
MAPK2	2.8E-03	7.3E-23	2.1E-03	1.9E-02	2.9E-03	1	1	no
MAPK3	5.3E-03	1.6E-21	2.7E-03	1.5E-02	2.5E-03	2	3	no
MAPK4	3.1E-02	5.6E-23	3.5E-02	1.6E-02	2.4E-03	5	2	no
MAT	5.8E-02	1.0E-17	8.0E-02	3.5E-02	2.0E-03	1	1	no
MDD	5.3E-02	4.1E-02	5.0E-18	4.7E-03	3.4E-03	2	1	yes
miR393	5.4E-04	9.3E-23	3.1E-22	9.8E-03	2.9E-03	1	2	no
MKS1	1.1E-02	2.3E-23	1.1E-02	2.3E-02	2.7E-03	2	1	no
MSK	5.2E-02	1.2E-17	3.7E-02	2.1E-02	2.0E-03	1	1	no
MTS/HMT	3.4E-02	8.5E-04	1.9E-02	3.1E-02	2.0E-03	1	1	no
MYC2	4.7E-03	2.8E-22	5.6E-03	1.6E-02	3.1E-03	2	3	no
NDR1	5.5E-06	3.0E-23	4.1E-06	1.5E-02	2.7E-03	1	1	no
NPR1	7.6E-03	2.9E-19	1.6E-03	2.7E-02	1.8E-03	3	3	no
OPR	2.8E-03	3.7E-23	2.6E-03	3.4E-02	5.8E-03	1	1	no
OSTIK	5.8E-03	1.2E-03	3.8E-03	7.8E-03	2.4E-03	1	2	yes
PAD4/EDS1	2.1E-03	5.7E-22	7.0E-03	1.7E-02	2.5E-03	3	3	no
PCT	7.7E-03	1.9E-22	6.7E-03	7.2E-03	4.2E-03	1	1	no
PDF1.2	0.0E+00	1.7E-25	0.0E+00	2.0E-02	2.4E-03	0	3	no
PED	3.0E-02	1.0E-19	3.6E-02	2.3E-02	3.4E-03	1	1	no

PES	3.7E-02	1.9E-18	3.5E-02	1.7E-02	3.4E-03	1	1	no
PhyB	1.1E-05	8.0E-21	1.7E-06	3.5E-02	1.1E-03	1	1	no
PPS	3.4E-02	3.8E-17	3.8E-02	1.2E-02	3.4E-03	1	1	no
PR1	3.7E-03	1.6E-23	4.2E-03	3.8E-02	5.1E-03	1	7	no
PSP	3.7E-02	1.8E-02	3.2E-03	7.3E-03	3.6E-03	2	1	yes
Pst DC3000	1.8E-01	6.2E-02	1.2E-16	7.9E-03	2.7E-03	8	1	yes
RbohD	6.1E-06	2.0E-21	1.2E-05	9.6E-03	5.4E-23	1	1	no
RESISTANCE	0.0E+00	2.7E-26	0.0E+00	2.8E-02	2.8E-03	0	3	no
ROS	2.9E-06	5.6E-22	3.6E-05	1.5E-02	2.0E-03	1	3	no
SA	8.2E-03	3.1E-19	1.5E-02	1.3E-02	2.0E-03	5	11	no
SCF Com	0.0E+00	6.2E-27	0.0E+00	3.2E-02	1.1E-03	0	1	no
SCF. Com	3.2E-02	6.0E-03	2.2E-02	5.8E-02	6.8E-03	1	1	no
SCF-COII	1.3E-02	5.7E-24	1.8E-02	1.3E-02	3.2E-03	1	2	no
Stom. Clos.	5.3E-04	2.6E-24	1.9E-03	1.4E-02	4.5E-03	1	1	no
TGA-TF	3.8E-03	2.7E-24	2.6E-03	3.0E-02	3.2E-03	2	4	no
TIRI	0.0E+00	8.3E-27	0.0E+00	1.4E-02	2.7E-03	0	4	no
T-LRR	1.3E-02	5.1E-03	6.8E-09	6.7E-03	3.4E-03	2	1	yes
TMO/TPM/RPT	2.8E-02	9.2E-03	3.5E-03	6.6E-03	3.1E-03	2	1	yes
WRK11	1.0E-02	5.6E-03	6.8E-04	7.0E-03	3.8E-03	2	1	yes
WRK17	1.0E-02	5.4E-03	2.5E-20	6.5E-03	3.4E-03	2	1	yes
WRK25-33	7.5E-07	3.4E-23	1.4E-06	1.5E-02	2.6E-11	1	2	no
WRK70	1.0E-03	2.1E-23	1.0E-03	3.4E-03	1.8E-03	3	2	no
WRKY62	5.7E-08	1.9E-25	8.6E-08	3.3E-02	2.1E-03	1	1	no
ZEO/XDH	6.1E-03	2.3E-18	4.8E-03	4.5E-02	3.4E-03	1	1	no

Supplementary Table 6: *A. thaliana* root stem cell niche

A manually modelled network (from Figure 3B in the source paper (see article), thus not including predicted interactions) describing dynamics in the *Arabidopsis thaliana* stem cell niche.

In this network we chose not to add loops to the input nodes to focus the analysis on the states converges to independently of external stimuli.

Node name	TC	VC	DC	TC-Sens.	VC-Sens.	Outgoing	Incoming	Loop
ACR4	1.4E-04	1.8E-17	1.2E-04	6.0E-02	2.8E-02	1	1	no
ARFa	6.9E-05	1.6E-17	8.0E-05	7.7E-02	1.9E-16	1	1	no
ARFi	2.7E-06	3.6E-17	3.0E-06	7.7E-02	1.7E-16	1	1	no
Auxin	2.4E-01	2.3E-16	2.4E-01	1.8E-17	7.6E-18	1	0	no
CLE40	1.6E-01	7.4E-02	7.1E-04	6.0E-02	2.8E-02	3	1	yes
IAA	1.7E-16	8.3E-17	1.3E-16	7.7E-02	1.8E-16	2	1	no
JKD	1.9E-02	9.0E-03	4.4E-03	2.6E-02	6.6E-03	3	4	yes
MGP	3.0E-02	5.8E-21	3.0E-02	3.3E-02	8.7E-04	1	4	yes
mR165	1.1E-03	3.1E-04	1.1E-03	1.5E-02	6.6E-03	2	3	yes
PHB	4.0E-03	6.2E-04	6.2E-03	1.6E-02	6.8E-03	2	1	no
SCR	4.3E-02	8.4E-03	3.2E-02	2.6E-02	6.6E-03	4	3	yes
SHR	8.9E-02	4.2E-02	1.0E-02	5.8E-02	2.7E-02	6	1	yes
WOX5	0.0E+00	3.3E-18	0.0E+00	5.7E-02	2.4E-02	0	6	no

Supplementary Table 7: *S. pombe* (fission yeast) cell cycle

A manually modelled network (from Figure 1 B in the source article, see article) describing the sequence of cell cycle activation patterns in Schizosaccharomyces pombe.

Node name	TC	VC	DC	TC-Sens.	VC-Sens.	Outgoing	Incoming	Loop
Cdc2/Cdc13	5.5E-02	3.4E-02	1.3E-03	2.5E-03	7.2E-14	6	4	no
Cdc2_Tyr15	5.0E-02	2.2E-02	3.0E-02	8.5E-02	2.0E-08	1	2	no
Cdc25	8.4E-02	2.4E-02	6.0E-02	8.0E-02	7.3E-04	2	3	yes
Cig1/Cdc2	1.4E-02	6.9E-03	4.0E-03	2.2E-17	5.1E-18	2	1	no
Cig2/Cdc2	1.4E-02	5.2E-03	4.0E-03	2.1E-17	5.2E-18	2	1	no
Clp1	2.5E-01	1.3E-02	2.2E-01	7.7E-02	2.2E-10	2	2	no
PP	3.7E-01	2.7E-02	3.3E-01	1.5E-01	3.9E-12	5	2	no

Puc1/Cdc2	1.2E-02	5.6E-03	4.0E-03	2.1E-17	5.1E-18	2	1	no
Rum1	1.9E-02	9.2E-03	7.3E-03	1.9E-01	5.7E-02	2	6	yes
Start	4.2E-02	2.7E-02	1.3E-02	7.4E-19	1.8E-19	3	0	no
Ste9	1.9E-02	9.7E-03	1.0E-02	2.0E-01	5.8E-02	2	6	yes
Wee1	7.2E-02	2.4E-02	5.3E-02	2.2E-01	9.3E-02	2	3	yes

Supplementary Table 8: *S. cerevisiae* (budding yeast)

A network describing the interaction of transcription factors in Saccharomyces cerevisiae compiled from data from the YEASTRACT database (accessed on 28.03.2014). The list of transcription factors with known targets was extracted from the file "RegulationTwoColumnTable_Documented_2013927.tsv.gz". Interactions were then obtained by querying the database for regulations proved by "Only Expression evidence" and "TF acting as activator" and "TF acting as inhibitor", respectively. To focus on regulatory processes and make the calculations computationally feasible, transcriptions factors which do not influence other transcription factors and proteins other than transcription factors were excluded from the network. For the values in brackets input loops were added.

Node name	TC	VC	DC	TC-Sens.	VC-Sens.	Outgoing	Incoming	Loop
abf1	2.1E-02	1.6E-02	1.9E-02	7.6E-03	3.5E-03	9	1	yes
ace2	1.5E-03	3.3E-20	7.3E-04	1.7E-02	2.1E-18	2	2	no
adr1	1.2E-03	8.9E-18	2.4E-03	2.4E-05	7.1E-06	2	5	no
aft1	1.1E-03	8.7E-20	9.4E-04	1.0E-02	3.9E-21	2	2	yes
aft2	9.5E-03	3.9E-21	1.0E-02	2.0E-02	5.7E-04	1	1	no
arg81	1.4E-03	4.4E-34	1.4E-03	1.4E-03	4.4E-34	1	1	yes
aro80	2.2E-20	4.3E-20	7.9E-19	3.1E-18	7.4E-19	2	2	yes
ash1	3.1E-05	2.4E-21	8.7E-08	5.5E-03	1.8E-03	2	4	no
cad1	1.1E-02	7.4E-03	1.0E-02	6.7E-03	3.3E-03	3	1	yes
cat8	0.0E+00	4.1E-23	0.0E+00	8.0E-03	9.8E-04	0	2	no
cbf1	1.3E-02	4.5E-04	9.2E-03	8.8E-03	1.9E-03	8	2	yes
cin5	3.6E-05	1.3E-20	1.7E-04	2.1E-03	2.0E-05	5	9	yes
dal80	4.7E-07	7.7E-23	3.7E-07	8.7E-05	1.3E-12	2	5	yes
dal81	0.0E+00	2.3E-23	0.0E+00	2.2E-03	3.5E-03	0	1	no
dal82	8.6E-03	4.6E-03	3.0E-04	6.1E-03	3.3E-03	2	1	yes
dig1	0.0E+00	2.8E-22	0.0E+00	3.9E-03	8.1E-20	0	3	no
fhl1	9.9E-03	5.7E-03	1.7E-05	7.6E-03	3.1E-03	6	1	yes
fkh1	1.4E-02	5.4E-03	3.3E-06	8.0E-03	3.1E-03	3	1	yes
fkh2	1.0E-02	1.9E-19	1.0E-02	9.8E-03	7.8E-19	2	1	no
flo8	1.9E-07	8.8E-22	2.9E-07	1.5E-18	7.2E-19	2	2	no
gal4	1.4E-04	3.9E-22	1.4E-04	3.9E-18	1.1E-18	1	2	no
gat1	6.2E-07	6.8E-20	1.3E-06	5.6E-04	7.5E-06	2	11	yes
gcn4	3.5E-04	7.9E-04	4.0E-05	7.8E-03	9.4E-09	12	3	no
gcr2	7.8E-03	2.9E-03	4.5E-08	7.8E-03	2.9E-03	2	1	yes
gln3	3.8E-09	2.4E-17	1.2E-08	9.1E-06	1.2E-10	5	4	yes
gzf3	8.4E-09	1.7E-22	9.9E-09	1.9E-03	8.1E-07	1	1	no
hal9	7.1E-03	5.7E-03	1.1E-03	7.1E-03	4.5E-03	2	1	yes
hap1	1.4E-04	1.0E-23	5.4E-06	6.8E-04	1.8E-04	3	7	yes
hap2	7.5E-03	2.4E-03	9.9E-04	7.5E-03	2.4E-03	2	1	yes
hap4	1.6E-02	1.6E-04	1.6E-02	1.4E-05	4.7E-07	1	10	no
hsf1	1.1E-02	5.0E-03	1.4E-03	7.6E-03	4.3E-03	3	1	yes
ifh1	8.0E-03	3.9E-03	9.8E-04	8.0E-03	3.9E-03	1	1	yes
ime1	7.0E-10	3.1E-23	3.0E-11	7.7E-08	1.6E-08	1	5	yes
ino2	0.0E+00	4.9E-22	0.0E+00	5.1E-18	1.2E-18	0	1	no
ino4	4.9E-05	2.8E-19	4.9E-05	4.1E-18	9.6E-19	4	2	yes
ixr1	1.7E-07	3.9E-23	1.5E-07	2.8E-04	6.5E-20	2	8	yes
leu3	4.4E-05	1.2E-18	3.7E-05	1.5E-07	9.8E-11	1	3	no
mbp1	1.0E-02	4.9E-03	1.1E-03	7.6E-03	3.7E-03	3	1	yes

mcm1	9.2E-03	3.9E-03	7.2E-05	8.4E-03	3.5E-03	6	1	yes
met28	3.0E-08	9.0E-22	2.9E-08	3.3E-03	5.7E-04	1	12	yes
met31	7.3E-03	3.3E-03	2.0E-03	7.3E-03	3.3E-03	2	1	yes
met32	1.5E-05	2.7E-22	4.9E-05	2.4E-03	2.9E-04	1	3	no
met4	1.4E-03	1.6E-04	8.1E-04	8.7E-03	3.8E-03	5	4	yes
mga1	5.8E-06	3.5E-21	3.0E-06	1.7E-03	8.5E-04	1	10	yes
mig1	7.2E-20	2.0E-19	2.2E-19	4.0E-18	1.2E-18	4	2	yes
mot3	0.0E+00	3.7E-23	0.0E+00	1.6E-03	1.6E-04	0	4	no
msn1	0.0E+00	3.0E-23	0.0E+00	2.0E-02	5.7E-04	0	1	no
msn2	1.0E-02	1.4E-16	9.3E-03	2.9E-03	7.3E-04	28	6	yes
msn4	2.6E-03	6.8E-18	3.3E-04	6.6E-04	1.8E-04	5	10	yes
ndt80	0.0E+00	4.9E-22	0.0E+00	6.2E-19	2.3E-19	0	1	no
nrg1	6.4E-06	9.0E-23	5.3E-06	2.7E-03	6.0E-04	1	9	no
oaf1	1.5E-02	7.9E-03	1.0E-02	7.5E-03	2.7E-03	4	1	yes
oaf3	7.5E-03	4.1E-03	6.5E-19	7.5E-03	4.1E-03	1	1	yes
pdr1	7.7E-06	1.8E-17	9.2E-06	5.5E-03	1.1E-05	5	3	no
pdr3	3.8E-03	1.4E-19	3.4E-03	4.0E-03	3.9E-05	4	5	yes
phd1	1.9E-06	1.6E-23	3.5E-06	1.7E-04	3.3E-05	2	14	yes
pho2	8.0E-03	4.1E-03	9.8E-04	8.0E-03	4.1E-03	2	1	yes
pho4	3.1E-03	6.6E-04	1.3E-03	8.3E-03	3.1E-03	2	4	no
pip2	1.9E-05	5.7E-17	1.9E-05	3.8E-03	2.7E-03	1	3	yes
put3	0.0E+00	2.0E-22	0.0E+00	6.0E-05	3.8E-06	0	4	no
rap1	2.1E-02	3.2E-04	2.0E-02	3.2E-18	6.5E-19	17	2	yes
rds1	2.5E-03	0.0E+00	2.6E-03	5.0E-03	1.2E-04	1	3	yes
rds2	1.2E-02	5.4E-03	1.0E-03	7.1E-03	3.3E-03	3	1	yes
reb1	4.7E-04	1.6E-24	1.1E-03	1.3E-03	3.7E-04	1	4	yes
rfx1	1.7E-07	1.6E-20	3.1E-08	2.9E-18	1.1E-18	3	2	yes
rgm1	0.0E+00	7.1E-23	0.0E+00	6.6E-19	2.3E-19	0	1	no
rgt1	0.0E+00	3.5E-22	0.0E+00	6.4E-19	2.2E-19	0	1	no
rlm1	0.0E+00	4.1E-22	0.0E+00	3.1E-03	3.3E-03	0	1	no
rme1	0.0E+00	6.4E-23	0.0E+00	7.4E-03	1.3E-04	0	3	no
rox1	2.3E-07	6.2E-21	5.9E-08	6.9E-05	5.7E-06	5	13	yes
rpn4	3.4E-02	1.5E-18	3.2E-02	2.9E-03	7.9E-04	6	5	no
rtg3	0.0E+00	3.0E-23	0.0E+00	1.2E-02	2.4E-03	0	4	no
sfp1	1.2E-03	3.1E-04	8.1E-04	4.1E-18	1.2E-18	4	2	no
sip4	0.0E+00	3.0E-22	0.0E+00	7.4E-03	2.6E-03	0	3	no
skn7	2.6E-05	1.3E-18	2.3E-04	2.9E-03	1.4E-03	8	2	no
sko1	1.5E-02	9.2E-03	4.3E-03	6.7E-03	4.3E-03	8	1	yes
smp1	8.8E-04	6.4E-25	2.8E-04	1.3E-03	2.1E-04	1	5	yes
sok2	1.4E-02	9.8E-04	1.3E-02	3.5E-03	9.8E-04	26	5	yes
spt23	1.4E-03	2.8E-28	1.4E-03	1.4E-03	4.7E-34	2	1	yes
stb5	5.7E-03	8.2E-24	5.8E-03	5.0E-03	7.2E-05	2	2	yes
ste12	5.0E-03	1.5E-16	6.4E-03	6.0E-19	2.6E-19	36	4	yes
stp1	3.2E-04	1.1E-18	6.4E-10	2.2E-03	3.5E-03	1	1	no
sum1	1.2E-13	3.7E-21	6.3E-14	5.2E-04	4.8E-20	2	3	yes
sut1	5.0E-04	1.9E-21	6.5E-04	1.1E-03	1.1E-04	2	5	yes
swi4	9.3E-03	1.1E-19	9.3E-03	1.6E-03	3.6E-04	11	7	yes
swi5	7.7E-05	3.8E-17	1.2E-04	6.3E-03	2.2E-03	3	8	no
swi6	3.1E-05	3.2E-19	5.6E-05	6.5E-19	2.3E-19	3	1	no
tec1	1.6E-03	1.1E-16	4.3E-04	6.3E-19	3.8E-19	16	6	yes
thi2	8.4E-03	3.7E-03	8.9E-22	8.4E-03	3.7E-03	1	1	yes
tos8	0.0E+00	2.5E-23	0.0E+00	2.9E-03	1.1E-03	0	11	no
tup1	1.7E-02	9.6E-03	5.4E-03	6.9E-03	3.9E-03	6	1	yes
tye7	2.5E-05	4.2E-04	2.6E-05	1.6E-03	9.7E-05	1	15	no
ume6	8.3E-04	2.6E-21	8.6E-06	3.6E-03	1.6E-03	2	3	no
xbp1	5.9E-06	8.6E-23	2.5E-08	1.9E-03	3.8E-04	1	9	no
yap1	3.2E-02	9.3E-17	2.8E-02	1.0E-02	4.4E-04	15	4	yes
yap6	1.2E-03	2.4E-21	1.2E-03	2.0E-03	4.4E-04	2	5	no
yap7	1.4E-03	3.6E-34	1.4E-03	1.4E-03	3.6E-34	1	1	yes
yhp1	0.0E+00	2.0E-22	0.0E+00	1.2E-03	2.4E-04	0	6	no
yox1	2.7E-06	1.7E-20	3.0E-06	8.5E-03	4.3E-05	2	3	no
yrm1	1.2E-03	7.8E-21	1.2E-03	1.0E-02	2.2E-03	1	2	yes
yrr1	2.8E-08	1.2E-22	8.2E-09	3.0E-03	6.1E-06	1	2	yes
zap1	3.9E-20	2.4E-20	4.6E-21	3.8E-18	1.5E-18	2	2	yes

Supplementary Table 9: *P. aeruginosa*

A network describing the interaction of transcription factors in *Pseudomonas aeruginosa* modeled from data by Galán-Vásquez et al. The interactions were taken from the sheet "network data" from the additional file "2042-5783-1-3-s1.xls", colums "Regulator (TF or sigma)" and "Target gene". Genes which do not have regulatory influence themselves were again omitted. For the values in brackets input loops were added.

Node	TC	VC	DC	TC-Sens.	VC-Sens.	Outgoing	Incoming	Loop
agmr	0.0E+00	3.2E-21	0.0E+00	2.2E-02	4.1E-03	0	1	no
agur	8.3E-03	4.8E-03	4.6E-03	8.3E-03	4.8E-03	1	1	yes
algq	3.9E-02	2.1E-02	2.4E-03	9.0E-03	4.1E-03	4	1	yes
algr	1.9E-04	1.9E-04	6.4E-07	1.2E-02	1.9E-03	2	3	yes
algr3	9.2E-03	3.5E-03	6.1E-04	8.3E-03	3.2E-03	2	1	yes
algr4	1.0E-02	6.5E-03	8.9E-06	8.3E-03	4.1E-03	2	1	yes
algu	3.5E-03	7.0E-17	4.3E-03	5.5E-04	7.2E-05	6	6	yes
algw	9.4E-03	4.7E-03	1.2E-03	8.7E-03	4.6E-03	2	1	yes
algz	5.5E-05	3.4E-17	3.9E-05	2.6E-03	1.9E-05	2	3	yes
ampr	8.9E-03	6.5E-03	1.2E-03	8.3E-03	4.6E-03	3	1	yes
anr	1.8E-02	5.2E-03	2.3E-02	1.0E-02	3.8E-03	3	2	yes
argr	9.4E-03	3.9E-03	1.1E-03	9.4E-03	3.9E-03	1	1	yes
bexr	9.9E-03	5.3E-03	2.3E-03	9.9E-03	5.3E-03	1	1	yes
bqsr	7.6E-03	3.7E-03	1.8E-18	7.6E-03	3.7E-03	1	1	yes
bqss	9.0E-03	4.4E-03	2.3E-03	9.0E-03	4.4E-03	1	1	yes
cbrb	8.5E-03	4.6E-03	1.1E-03	8.5E-03	4.6E-03	1	1	yes
cysb	1.6E-03	4.3E-34	1.6E-03	1.6E-03	4.3E-34	1	1	yes
dnr	7.6E-03	4.2E-03	1.8E-03	1.9E-02	6.4E-03	3	3	yes
exsa	4.8E-03	8.1E-17	5.8E-03	8.8E-03	2.4E-04	2	5	yes
exsd	8.3E-03	1.2E-17	7.1E-03	7.7E-03	6.7E-04	1	1	no
fhpr	4.8E-04	7.8E-22	3.3E-05	1.2E-03	5.9E-04	1	3	yes
fleq	6.4E-02	3.9E-17	6.8E-02	7.9E-03	3.4E-03	1	4	no
flgm	3.0E-03	7.0E-21	3.0E-03	3.9E-03	1.7E-20	1	1	no
flia	7.5E-03	4.5E-21	7.5E-03	6.6E-03	3.2E-20	1	1	no
fpvi	0.0E+00	2.0E-21	0.0E+00	3.1E-02	4.1E-03	0	1	no
fpvr	2.1E-03	4.8E-20	3.4E-03	2.9E-02	4.1E-03	2	1	no
fur	2.5E-02	1.3E-17	2.6E-02	1.7E-02	3.7E-03	5	1	no
gaca	7.4E-03	3.2E-03	1.4E-06	7.4E-03	3.2E-03	2	1	yes
gacs	8.7E-03	3.7E-03	1.1E-03	8.7E-03	3.7E-03	1	1	yes
gbur	2.3E-03	2.7E-17	9.2E-03	1.8E-02	4.1E-03	1	1	no
glpr	2.1E-03	1.2E-19	1.8E-11	2.0E-02	4.1E-03	1	1	no
gpur	9.2E-03	4.6E-03	1.1E-03	9.2E-03	4.6E-03	1	1	yes
hu	8.2E-03	5.3E-03	1.2E-03	8.0E-03	5.1E-03	2	1	yes
ihf	8.5E-03	3.2E-03	9.2E-06	8.5E-03	3.2E-03	2	1	yes
lasr	1.3E-02	5.6E-04	1.1E-02	1.3E-03	1.5E-03	6	6	no
metr	8.7E-03	5.1E-03	1.1E-03	8.7E-03	5.1E-03	1	1	yes
mexr	1.9E-02	2.0E-03	1.7E-02	1.9E-03	1.9E-03	2	1	no
mext	1.1E-02	5.9E-04	9.7E-03	1.9E-02	3.0E-03	2	2	yes
mexz	0.0E+00	3.8E-21	0.0E+00	9.0E-03	4.1E-03	0	1	no
muca	9.3E-05	2.4E-17	1.3E-04	5.8E-04	8.0E-05	1	1	no
mucb	2.7E-04	3.1E-17	2.6E-06	5.8E-04	8.0E-05	1	1	no
mucc	9.9E-03	2.1E-03	2.9E-03	9.0E-03	2.1E-03	2	1	yes
mucd	1.0E-02	4.5E-03	1.4E-03	9.7E-03	4.4E-03	2	1	yes
mvat	8.0E-03	3.7E-03	2.3E-03	8.0E-03	3.7E-03	1	1	yes
mvfr	4.0E-08	1.2E-21	8.5E-08	2.5E-03	2.7E-04	1	3	yes
mvta	9.3E-03	5.9E-03	1.3E-03	9.2E-03	5.7E-03	2	1	yes
nalc	0.0E+00	2.7E-20	0.0E+00	1.3E-02	2.0E-03	0	1	no
narl	0.0E+00	2.7E-21	0.0E+00	1.0E-02	3.8E-03	0	2	no
nfxb	1.6E-03	4.4E-34	1.6E-03	1.6E-03	4.4E-34	1	1	yes
ospr	8.7E-03	3.4E-03	2.8E-19	8.7E-03	3.4E-03	1	1	yes
pa0779	9.2E-03	3.9E-03	1.7E-03	8.5E-03	3.4E-03	2	1	yes
pa3697	8.3E-03	5.3E-03	2.3E-20	8.3E-03	5.3E-03	1	1	yes
pa5471	1.8E-02	8.3E-03	3.4E-03	9.0E-03	4.1E-03	2	1	yes

pchr	9.5E-04	6.1E-26	3.3E-04	5.5E-03	7.8E-04	1	2	yes
pfer	0.0E+00	6.7E-21	0.0E+00	1.8E-02	4.1E-03	0	1	no
phhr	1.6E-03	3.9E-34	1.6E-03	1.6E-03	3.9E-34	1	1	yes
phop	2.6E-02	1.8E-02	8.0E-03	8.7E-03	5.3E-03	3	1	yes
phoq	2.5E-02	6.5E-04	1.6E-02	8.7E-03	5.3E-03	2	1	no
pila	4.5E-03	1.0E-22	4.7E-03	4.9E-03	1.9E-04	1	3	yes
pilr	8.5E-03	4.7E-03	8.8E-06	8.3E-03	4.6E-03	2	1	yes
pmra	3.9E-03	2.8E-03	1.1E-03	1.3E-02	3.9E-03	2	2	yes
pprb	9.0E-05	8.5E-19	1.0E-04	9.9E-03	3.5E-08	2	2	no
ppyr	9.4E-03	4.1E-03	2.4E-19	9.4E-03	4.1E-03	1	1	yes
pqrr	1.0E-02	5.1E-03	3.4E-03	1.0E-02	5.1E-03	1	1	yes
psdr	9.9E-03	3.9E-03	1.1E-03	9.9E-03	3.9E-03	1	1	yes
psra	1.9E-03	9.8E-21	3.1E-03	1.6E-03	4.1E-34	3	1	yes
ptxr	5.3E-05	2.2E-22	2.1E-05	5.7E-03	6.8E-04	1	4	no
ptxs	9.4E-03	5.0E-23	8.0E-03	5.1E-03	7.8E-05	2	4	yes
pvds	1.0E-04	1.0E-19	7.9E-05	2.1E-03	5.8E-08	2	3	no
qscr	8.3E-03	5.3E-03	1.1E-03	8.3E-03	5.3E-03	2	1	yes
rcdb	8.5E-03	5.1E-03	1.6E-19	8.5E-03	5.1E-03	1	1	yes
rhlr	3.3E-04	3.0E-17	5.8E-05	6.5E-04	2.8E-04	3	7	yes
roca1	8.5E-03	3.4E-03	1.1E-03	8.5E-03	3.4E-03	1	1	yes
rocr	9.7E-03	4.6E-03	7.3E-20	9.7E-03	4.6E-03	1	1	yes
roxr	8.7E-03	4.6E-03	5.9E-19	8.7E-03	4.6E-03	1	1	yes
roxs	8.5E-03	4.6E-03	1.1E-03	8.5E-03	4.6E-03	1	1	yes
rpod	5.9E-03	6.8E-18	2.0E-04	9.0E-03	4.1E-03	2	1	no
rpon	8.3E-03	4.7E-03	1.2E-03	8.0E-03	4.4E-03	4	1	yes
rpos	2.6E-04	2.4E-17	3.4E-04	2.9E-03	2.8E-03	1	4	no
rsal	0.0E+00	3.6E-21	0.0E+00	3.3E-03	2.0E-04	0	2	no
toxr	0.0E+00	2.0E-20	0.0E+00	2.1E-03	1.3E-09	0	1	no
tpba	3.5E-03	3.5E-23	3.3E-03	4.3E-03	4.8E-04	1	3	yes
trpi	1.6E-03	4.6E-34	1.6E-03	1.6E-03	4.6E-34	1	1	yes
vfr	5.9E-02	2.7E-02	1.0E-02	9.4E-03	3.7E-03	5	1	yes
vqsm	1.5E-02	9.9E-03	7.8E-04	9.7E-03	4.1E-03	4	1	yes
vqsr	9.0E-03	2.1E-17	6.9E-03	3.0E-03	3.6E-03	1	2	no
vrei	9.2E-03	3.0E-03	2.3E-03	9.2E-03	3.0E-03	1	1	yes

Supplementary Table 10: *E. coli*

A network describing the interaction of transcription factors in *Escherichia coli* modeled from data from RegulonDB 8.5, specifically the first two columns in the file "network_tf_tf.txt" from the "Downloadable Experimental Datasets" section.

Node name	TC	VC	DC	TC-Sens.	VC-Sens.	Outgoing	Incoming	Loop
acrr	9.9E-04	2.4E-34	9.9E-04	9.9E-04	2.4E-34	1	1	yes
ada	1.5E-03	6.6E-22	1.6E-03	1.3E-18	3.0E-20	3	2	yes
adiy	1.0E-07	6.3E-25	3.2E-08	1.7E-03	2.5E-03	1	1	no
agar	9.9E-04	3.1E-34	9.9E-04	9.9E-04	3.1E-34	1	1	yes
aidb	1.7E-21	8.1E-26	1.9E-25	1.5E-03	3.2E-20	1	3	yes
alas	9.9E-04	1.7E-34	9.9E-04	9.9E-04	1.7E-34	1	1	yes
allr	4.7E-03	2.3E-03	4.8E-23	4.7E-03	2.3E-03	1	1	yes
alsr	5.2E-03	2.8E-03	1.4E-03	5.2E-03	2.8E-03	1	1	yes
arac	5.4E-22	3.5E-20	2.2E-22	3.5E-18	4.2E-20	2	3	yes
arca	2.5E-04	8.5E-17	6.9E-04	1.6E-05	5.1E-06	7	2	no
argp	2.2E-03	8.3E-25	2.3E-03	1.9E-03	3.2E-04	2	2	yes
argr	9.9E-04	2.4E-34	9.9E-04	9.9E-04	2.4E-34	1	1	yes
arsr	9.9E-04	2.6E-34	9.9E-04	9.9E-04	2.6E-34	1	1	yes
ascg	5.9E-03	3.0E-03	6.9E-04	5.9E-03	3.0E-03	2	1	yes
asnc	8.7E-04	0.0E+00	1.0E-03	1.1E-03	1.5E-04	1	2	yes
baer	8.8E-04	5.5E-04	9.0E-25	2.9E-03	1.3E-03	1	2	yes
basr	4.5E-03	2.8E-03	2.2E-06	4.4E-03	2.8E-03	3	1	yes
beti	4.4E-04	9.9E-26	1.9E-04	2.1E-03	5.1E-04	1	3	yes
cadc	8.5E-07	1.8E-24	2.7E-06	3.0E-03	8.9E-04	1	5	yes
cdar	4.8E-03	2.3E-03	2.1E-23	4.8E-03	2.3E-03	1	1	yes
chbr	0.0E+00	2.8E-25	0.0E+00	9.9E-04	2.4E-22	0	2	no
cpxr	8.0E-03	3.1E-03	2.1E-03	5.2E-03	1.8E-03	3	1	yes

cra	1.4E-03	1.5E-23	1.2E-03	1.8E-03	2.2E-03	7	1	no
crp	2.0E-02	3.0E-03	1.4E-02	2.3E-03	7.9E-22	41	3	yes
csgd	3.9E-08	1.1E-24	4.6E-08	1.7E-03	1.3E-03	1	12	yes
cspa	5.7E-03	2.5E-03	7.7E-23	5.7E-03	2.5E-03	1	1	yes
cusr	9.9E-04	5.5E-04	4.6E-22	2.5E-03	1.1E-03	1	2	yes
cynr	9.9E-04	2.3E-34	9.9E-04	9.9E-04	2.3E-34	1	1	yes
cysb	2.1E-24	9.5E-25	6.6E-24	9.9E-04	1.2E-19	1	2	yes
cytr	9.9E-04	3.0E-34	9.9E-04	9.9E-04	3.0E-34	1	1	yes
dan	5.5E-03	2.2E-03	6.9E-04	5.5E-03	2.2E-03	1	1	yes
dcur	2.1E-05	7.9E-21	3.1E-06	1.4E-03	4.5E-04	1	3	no
dhar	9.9E-04	1.9E-34	9.9E-04	9.9E-04	1.9E-34	1	1	yes
dnaa	1.0E-03	9.9E-27	1.1E-03	1.6E-03	9.0E-05	1	2	yes
dpia	5.7E-08	3.3E-20	8.6E-08	8.2E-05	2.4E-05	1	6	yes
dsdc	2.9E-04	4.4E-28	3.6E-04	9.3E-04	4.9E-04	1	2	yes
evga	5.7E-04	5.7E-04	8.7E-07	4.8E-03	2.6E-03	3	2	yes
exur	9.9E-04	4.7E-28	9.9E-04	9.9E-04	3.1E-34	2	1	yes
fadr	5.6E-03	2.8E-03	9.9E-04	5.4E-03	2.5E-03	2	1	yes
fhla	5.5E-04	7.1E-18	4.6E-06	3.9E-03	2.3E-03	2	4	yes
fis	2.1E-03	1.1E-04	2.3E-03	2.4E-03	1.9E-04	9	3	yes
flhdc	5.7E-03	2.5E-03	4.6E-06	5.7E-03	2.5E-03	2	1	yes
fliz	6.6E-03	8.0E-05	6.8E-03	1.8E-03	1.6E-03	3	4	no
fnr	1.2E-02	8.1E-05	1.0E-02	1.5E-03	6.1E-04	18	4	yes
fucr	4.9E-03	7.7E-04	3.9E-03	6.1E-03	2.4E-03	1	2	yes
fur	8.4E-03	6.3E-19	8.4E-03	2.0E-04	1.3E-05	7	4	yes
gade	2.6E-04	4.8E-20	2.6E-04	5.0E-03	2.5E-03	4	9	yes
gade-rcsb	5.4E-03	2.6E-03	6.7E-09	5.4E-03	2.6E-03	2	1	yes
gadw	3.1E-05	1.6E-20	4.3E-07	1.5E-04	1.1E-04	3	10	yes
gadx	3.7E-07	1.9E-21	1.3E-06	5.0E-05	2.0E-06	4	14	yes
galr	1.7E-03	5.0E-27	1.7E-03	2.1E-03	2.2E-22	2	2	yes
gals	1.0E-04	9.3E-25	1.0E-04	6.8E-04	2.5E-22	2	3	yes
gcva	9.9E-04	2.3E-34	9.9E-04	9.9E-04	2.3E-34	1	1	yes
glcc	3.2E-25	7.1E-23	1.1E-25	8.5E-04	7.0E-22	1	4	yes
gntr	9.9E-03	2.6E-03	3.3E-03	5.5E-03	1.8E-03	2	1	yes
gutm	4.3E-06	7.7E-20	3.7E-26	4.9E-03	1.6E-03	1	4	yes
gutr	1.0E-02	3.1E-03	2.7E-03	5.8E-03	1.8E-03	2	1	yes
hcar	9.9E-04	2.1E-34	9.9E-04	9.9E-04	2.1E-34	1	1	yes
hdfr	0.0E+00	2.8E-24	0.0E+00	1.7E-03	2.5E-03	0	1	no
hipab	5.6E-03	3.9E-03	1.6E-03	5.1E-03	3.3E-03	2	1	yes
hipb	2.5E-04	1.3E-23	1.8E-04	8.1E-04	7.2E-04	1	3	yes
h-ns	3.1E-02	2.0E-02	2.5E-02	5.2E-03	2.5E-03	17	1	yes
hyfr	1.9E-07	5.6E-19	1.9E-07	2.8E-03	5.1E-06	1	4	yes
iclr	2.2E-03	6.1E-26	1.9E-03	2.5E-03	3.6E-04	1	2	yes
idnr	6.6E-04	3.3E-04	3.0E-22	5.5E-03	1.5E-03	1	3	yes
ihf	9.4E-03	6.9E-03	1.9E-03	5.5E-03	2.2E-03	10	1	yes
ilvy	9.9E-04	2.2E-34	9.9E-04	9.9E-04	2.2E-34	1	1	yes
iscr	9.9E-04	3.1E-34	9.9E-04	9.9E-04	3.1E-34	1	1	yes
leuo	7.4E-04	8.8E-21	1.2E-05	5.3E-03	2.3E-03	2	4	yes
lexa	9.9E-04	1.8E-34	9.9E-04	9.9E-04	1.8E-34	1	1	yes
lldr	1.8E-03	0.0E+00	1.8E-03	1.9E-03	2.0E-06	1	2	yes
lrha	5.4E-03	1.7E-03	6.9E-04	5.4E-03	1.7E-03	1	1	yes
lrp	1.3E-03	9.1E-18	1.1E-03	6.9E-04	2.7E-04	4	4	yes
lsrr	2.3E-24	2.4E-25	2.0E-24	9.9E-04	9.2E-22	1	2	yes
lysr	9.9E-04	2.2E-34	9.9E-04	9.9E-04	2.2E-34	1	1	yes
mali	1.4E-24	2.7E-25	1.3E-26	9.9E-04	8.5E-22	1	3	yes
mara	2.9E-04	1.1E-23	7.6E-05	2.1E-03	1.3E-04	4	7	yes
marr	3.9E-03	1.9E-24	5.1E-03	2.1E-03	1.3E-04	2	7	yes
mata	7.8E-03	4.9E-03	2.1E-03	5.7E-03	3.0E-03	2	1	yes
maze	5.0E-04	2.5E-26	6.3E-04	8.7E-04	2.1E-04	1	5	yes
maze-mazf	6.4E-03	2.9E-03	8.8E-04	6.1E-03	2.8E-03	2	1	yes
melr	1.2E-21	3.6E-23	8.7E-22	4.6E-18	6.0E-21	2	3	yes
metj	7.1E-04	7.7E-25	7.1E-04	6.9E-03	1.2E-06	1	1	no
metr	2.3E-05	3.2E-30	2.3E-05	2.1E-03	3.8E-08	1	2	yes
mlc	3.5E-24	1.0E-24	1.4E-26	9.9E-04	7.4E-22	1	3	yes
mlra	6.0E-06	4.6E-23	6.8E-04	8.6E-03	1.9E-03	2	1	no
mngr	9.9E-04	2.4E-34	9.9E-04	9.9E-04	2.4E-34	1	1	yes
mode	5.5E-03	3.8E-03	1.9E-03	4.8E-03	2.9E-03	2	1	yes
mpra	9.9E-04	2.6E-34	9.9E-04	9.9E-04	2.6E-34	1	1	yes
mtlr	6.0E-26	3.5E-25	2.8E-25	8.5E-04	6.6E-22	1	4	yes
murr	4.8E-03	2.9E-03	7.5E-23	4.8E-03	2.9E-03	1	1	yes
nac	4.3E-04	1.8E-26	6.4E-04	7.7E-04	4.7E-04	2	4	yes

nagc	9.9E-04	1.4E-26	9.9E-04	9.9E-04	2.4E-34	2	1	yes
narl	3.2E-04	4.5E-17	4.3E-04	4.4E-03	2.0E-03	3	2	no
nemr	7.0E-05	8.2E-34	7.0E-05	1.4E-04	1.9E-33	1	2	yes
nhar	2.8E-04	6.9E-04	8.4E-25	5.2E-03	2.2E-03	1	2	yes
nikr	7.2E-04	7.7E-26	1.2E-03	1.2E-03	1.9E-04	1	3	yes
norr	3.6E-04	1.0E-29	2.2E-05	1.0E-03	4.9E-04	1	2	yes
nsrr	1.3E-02	7.9E-03	6.6E-03	6.1E-03	2.5E-03	6	1	yes
ntrc	5.8E-03	3.0E-03	8.3E-04	5.5E-03	2.5E-03	2	1	yes
ompr	1.3E-07	2.9E-25	2.0E-07	1.5E-03	6.7E-22	1	3	no
oxyr	1.5E-06	7.7E-17	1.9E-06	9.9E-04	7.2E-22	2	2	yes
pdhr	4.8E-24	6.2E-24	5.3E-24	1.2E-03	7.1E-22	1	3	yes
pepa	9.9E-04	2.4E-34	9.9E-04	9.9E-04	2.4E-34	1	1	yes
phob	9.4E-03	6.2E-03	8.2E-04	5.1E-03	2.2E-03	7	1	yes
phop	9.9E-04	3.4E-19	9.9E-04	2.7E-18	1.2E-19	7	2	yes
prpr	4.5E-24	2.7E-25	8.7E-25	1.3E-04	2.2E-22	1	4	yes
pspf	9.9E-04	2.3E-34	9.9E-04	9.9E-04	2.3E-34	1	1	yes
purr	1.8E-03	0.0E+00	1.8E-03	1.9E-03	5.1E-06	1	2	yes
puta	1.5E-05	1.2E-25	5.3E-05	4.5E-04	5.5E-05	1	3	yes
puur	1.4E-03	0.0E+00	1.4E-03	1.5E-03	1.7E-06	1	3	yes
qseb	5.9E-03	1.9E-03	6.9E-04	5.9E-03	1.9E-03	1	1	yes
rbsr	7.9E-25	2.9E-25	6.9E-24	9.9E-04	7.9E-22	1	2	yes
rcda	4.1E-03	3.5E-03	6.9E-04	4.1E-03	3.4E-03	2	1	yes
rcnr	9.9E-04	2.7E-34	9.9E-04	9.9E-04	2.7E-34	1	1	yes
rcsab	5.4E-03	3.2E-03	7.1E-04	4.6E-03	2.9E-03	2	1	yes
rcsb	5.1E-04	2.0E-17	5.7E-04	1.7E-03	2.5E-03	2	1	no
rcsb-bglj	6.3E-03	3.3E-03	2.9E-06	4.6E-03	2.2E-03	2	1	yes
relb	6.0E-04	1.7E-26	1.0E-03	1.1E-03	4.6E-04	1	2	yes
relb-rele	5.5E-03	2.8E-03	4.1E-04	5.0E-03	2.3E-03	2	1	yes
rhar	1.7E-03	6.3E-24	1.1E-04	2.1E-03	1.7E-21	2	3	yes
rhas	2.0E-03	2.9E-23	2.8E-03	2.1E-03	2.0E-21	2	3	yes
rob	1.8E-03	3.5E-27	2.0E-03	2.2E-03	6.4E-05	3	3	yes
rsta	3.5E-06	1.7E-24	3.0E-06	3.6E-18	1.5E-19	1	1	no
rutr	1.1E-03	3.6E-19	1.1E-03	9.9E-04	3.5E-34	4	1	yes
sdia	5.0E-03	2.2E-03	6.9E-04	5.0E-03	2.2E-03	2	1	yes
sgrr	9.9E-04	3.0E-34	9.9E-04	9.9E-04	3.0E-34	1	1	yes
soxr	8.7E-04	7.1E-20	7.8E-04	6.6E-04	1.2E-04	2	3	yes
soxs	6.5E-04	5.9E-17	8.1E-04	1.2E-03	1.5E-04	5	4	yes
stpa	4.9E-04	6.3E-22	1.2E-03	7.4E-04	1.1E-04	2	3	yes
tdca	1.9E-05	3.0E-24	6.6E-05	8.9E-04	1.0E-03	1	5	yes
tdcr	5.2E-03	3.5E-03	1.3E-03	4.6E-03	3.0E-03	2	1	yes
torr	9.9E-04	9.9E-23	9.9E-04	9.9E-04	2.4E-34	2	1	yes
trpr	9.9E-04	2.8E-34	9.9E-04	9.9E-04	2.8E-34	1	1	yes
tyrr	9.9E-04	2.6E-34	9.9E-04	9.9E-04	2.6E-34	1	1	yes
uidr	9.9E-04	2.8E-34	9.9E-04	9.9E-04	2.8E-34	1	1	yes
uxur	2.9E-22	4.3E-25	6.2E-24	5.3E-04	2.9E-22	1	3	yes
xylr	2.4E-10	5.7E-21	3.1E-10	7.7E-04	1.8E-08	1	4	yes
ydeo	7.9E-04	1.4E-22	2.7E-04	1.3E-03	7.2E-04	2	5	yes
yefm	4.0E-04	7.0E-30	3.6E-04	1.0E-03	3.8E-04	1	2	yes
yefm-yoeb	6.6E-03	2.3E-03	1.8E-03	5.9E-03	1.9E-03	2	1	yes
yeil	1.8E-04	8.6E-20	9.5E-07	5.5E-03	6.2E-04	1	4	yes
yiaj	3.3E-04	6.6E-32	1.2E-05	1.2E-03	4.4E-04	1	3	yes
yqji	1.8E-03	1.9E-26	1.3E-03	2.3E-03	1.6E-04	1	2	yes
zrar	5.1E-03	9.9E-04	3.3E-03	6.1E-03	2.0E-03	1	2	yes

Supplementary Figures: Other centralities in the biological networks

Supplementary Figure 1

a Total centrality and **b** dynamic centrality of nodes and connections in the T-helper network in a green-yellow-red colour scheme (0 to maximum controllability).

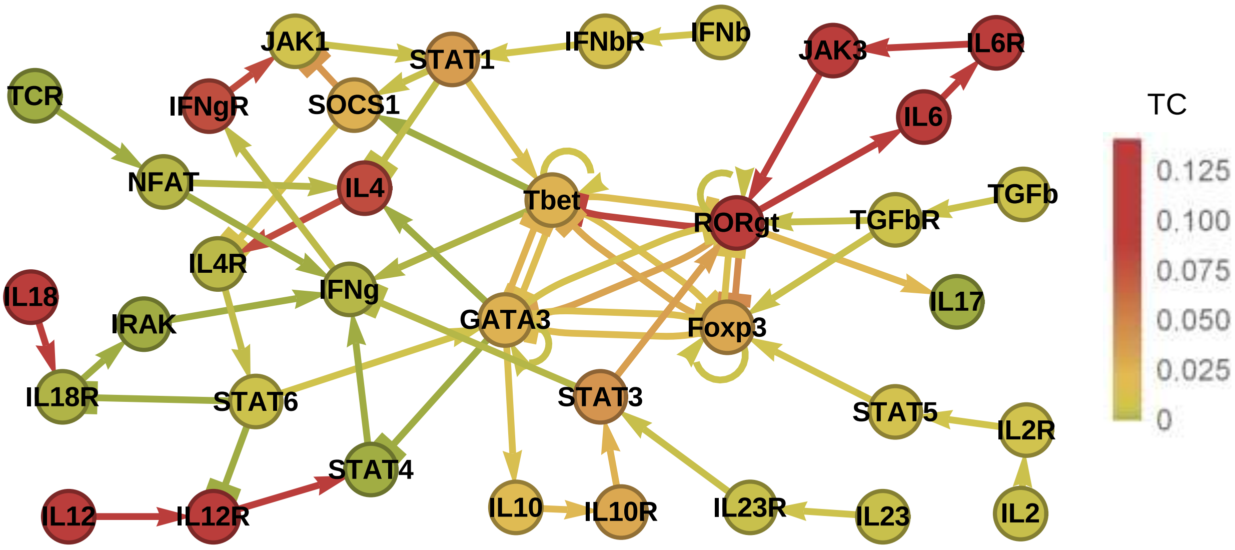

a

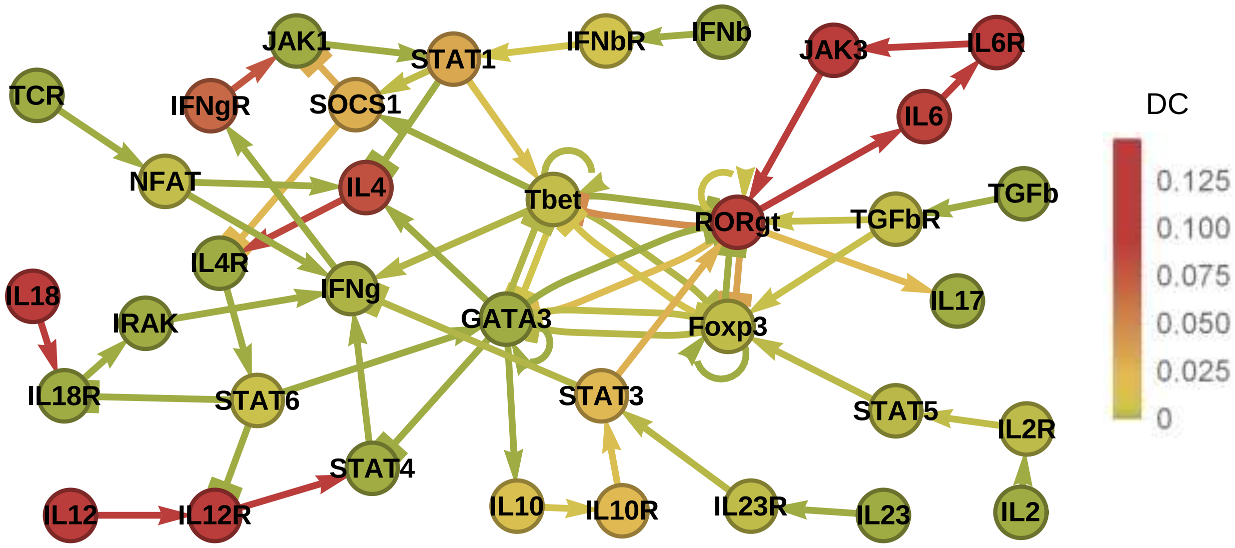

b

Supplementary Figure 2

a Value centrality and **b** dynamic centrality of nodes and connections in the chondrocyte network in a green-yellow-red colour scheme (from 0 to maximum controllability).

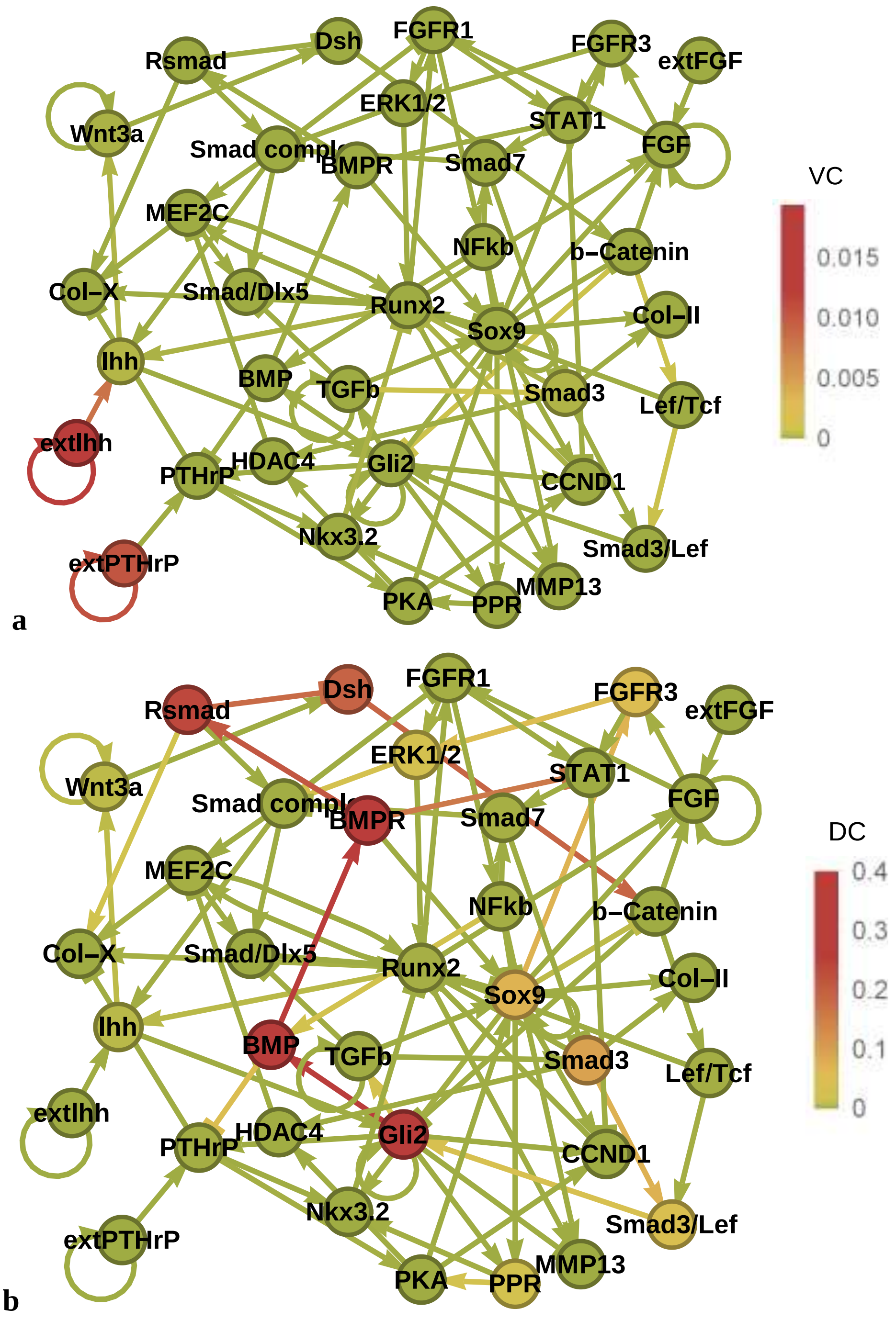

Supplementary Figure 3

a Value centrality, **b** dynamic centrality and **c** total centrality of nodes and connections in the A. thaliana immune response network in a green-yellow-red colour scheme (from 0 to maximum controllability).

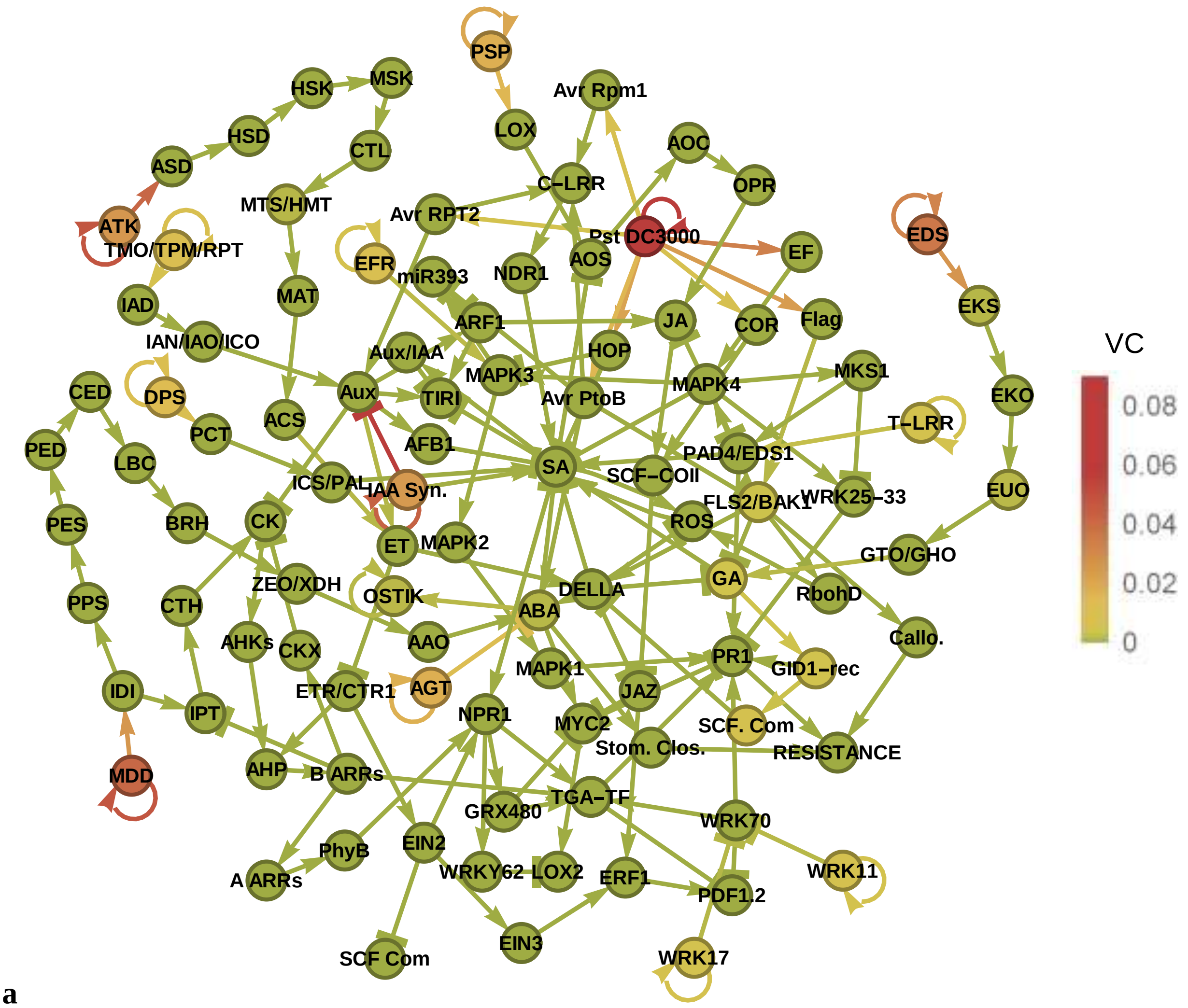

a

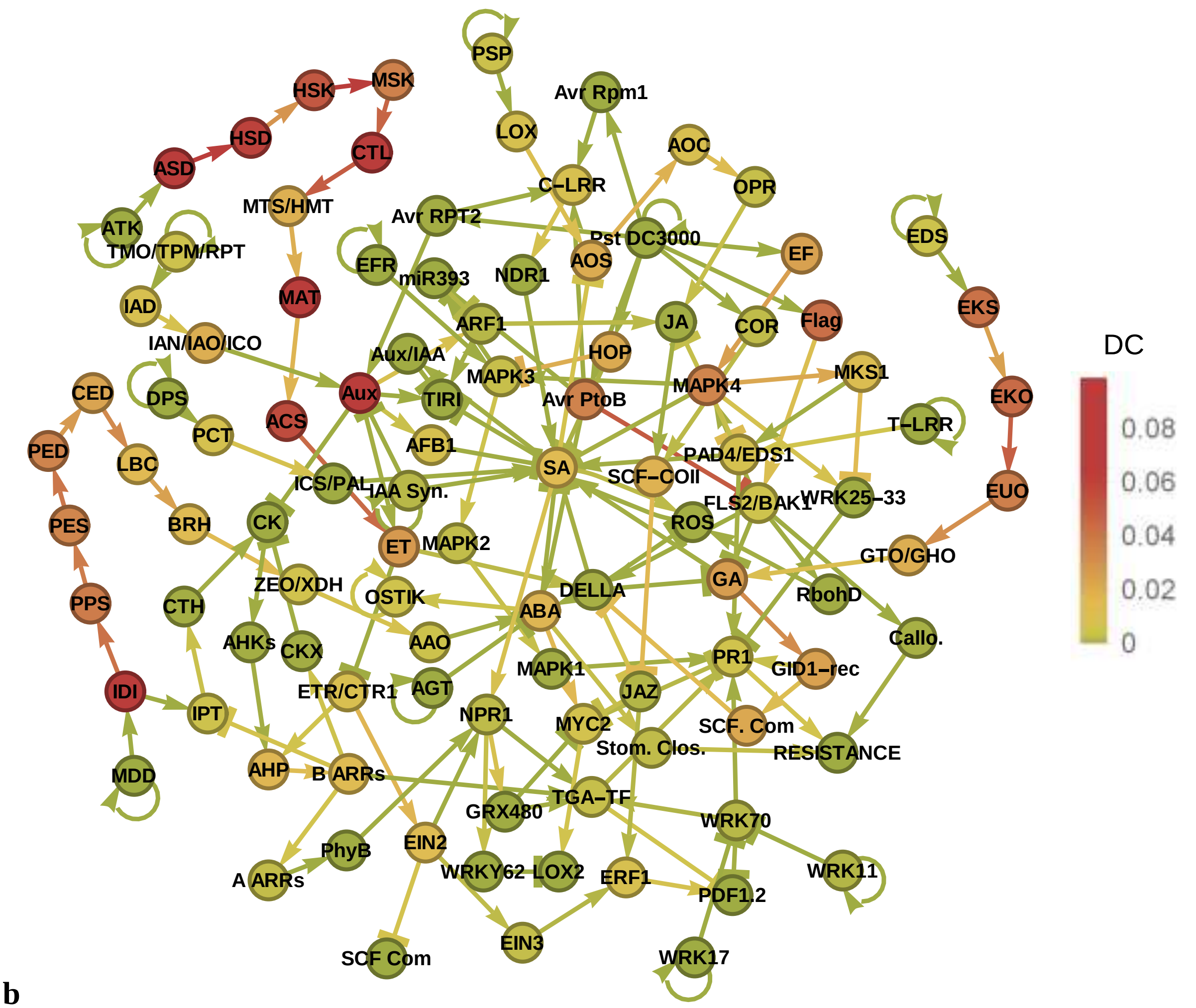
PSP
LOX
Avr Rpm1
C–LRR
AOC
OPR
HSK
MSK
HSD
CTL
ASD
ATK
TMO/TPM/RPT
MTS/HMT
Avr RPT2
Pst DC3000
EF
EDS
EFR
miR393
NDR1
AOS
MAT
IAD
ARF1
JA
COR
Flag
EKS
IAN/IAO/ICO
Aux/IAA
HOP
MAPK3
MAPK4
MKS1
CED
DPS
Aux
TIRI
Avr PtoB
ACS
EKO
PED
LBC
PCT
AFB1
T–LRR
PAD4/EDS1
SA
SCF–COII
ICS/PAL
IAA Syn.
FLS2/BAK1
WRK25–33
EUO
PES
BRH
CK
ROS
ET
MAPK2
GTO/GHO
GA
ZEO/XDH
DELLA
RbohD
PPS
CTH
OSTIK
ABA
Callo.
AHKs
CKX
AAO
PR1
MAPK1
GID1–rec
IDI
ETR/CTR1
AGT
JAZ
IPT
NPR1
MYC2
SCF. Com
Stom. Clos.
RESISTANCE
MDD
AHP
B ARRs
TGA–TF
GRX480
WRK70
PhyB
EIN2
A ARRs
WRKY62
LOX2
ERF1
WRK11
PDF1.2
EIN3
SCF Com
WRK17
DC
0.08
0.06
0.04
0.02
0

b

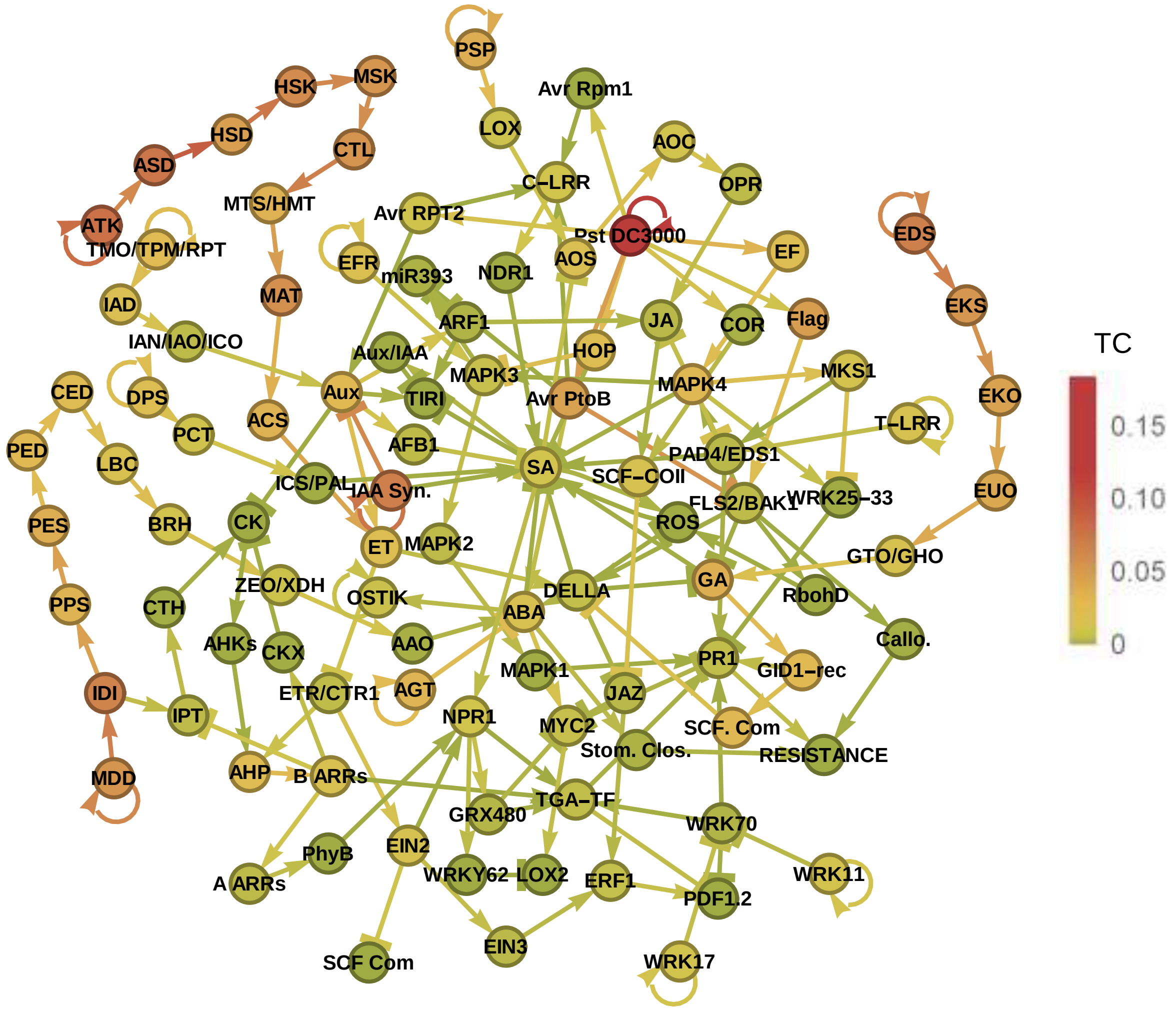

PSP
Avr Rpm1
LOX
AOC
OPR
C–LRR
Avr RPT2
Pst DC3000
EF
EDS
EKS
EKO
EUO
HSK
MSK
HSD
ASD
CTL
ATK
MTS/HMT
TMO/TPM/RPT
IAD
MAT
IAN/IAO/ICO
EFR
miR393
NDR1
AOS
ARF1
JA
COR
Flag
Aux/IAA
HOP
MAPK3
MAPK4
MKS1
Avr PtoB
CED
DPS
PCT
ACS
Aux
TIRI
AFB1
T–LRR
PED
LBC
SA
SCF–COII
PAD4/EDS1
ICS/PAL
IAA Syn.
FLS2/BAK1
WRK25–33
PES
BRH
CK
ROS
ET
MAPK2
GTO/GHO
GA
DELLA
RbohD
ZEO/XDH
OSTIK
ABA
PPS
CTH
AHKs
CKX
AAO
Callo.
MAPK1
PR1
GID1–rec
IDI
ETR/CTR1
AGT
JAZ
IPT
NPR1
MYC2
SCF. Com
Stom. Clos.
RESISTANCE
MDD
AHP
B ARRs
TGA–TF
GRX480
WRK70
PhyB
EIN2
A ARRs
WRKY62
LOX2
ERF1
PDF1.2
WRK11
EIN3
SCF Com
WRK17
TC
0.15
0.10
0.05
0

c

Supplementary Figure 4

a Total centrality, **b** value centrality and **c** dynamic centrality of nodes and connections in the *A. thaliana* root stem cell niche network in a green-yellow-red colour scheme (from 0 to maximum controllability).

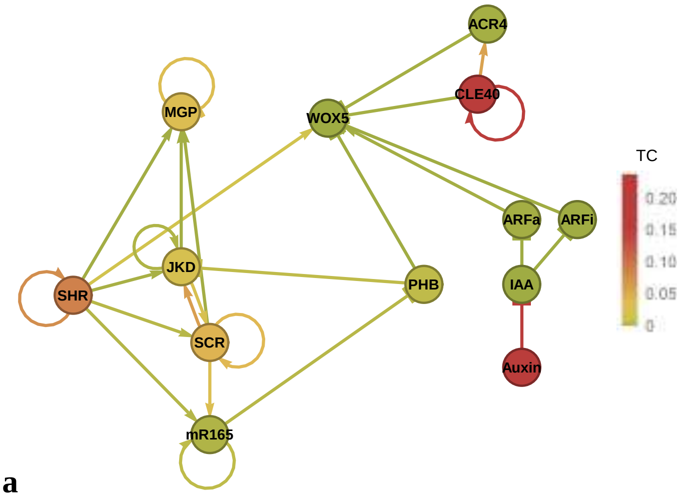

a

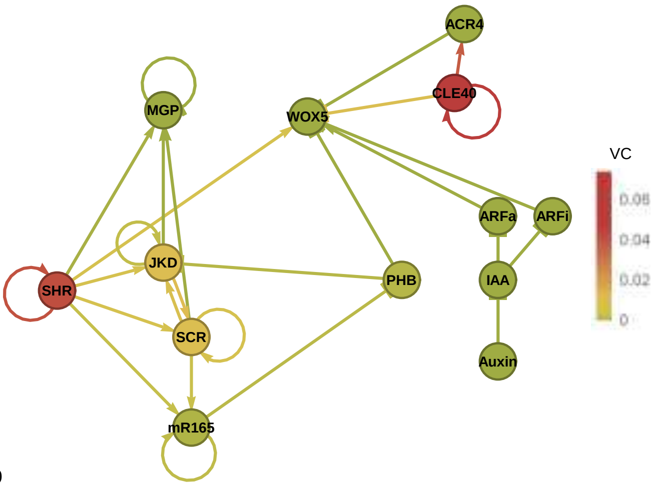

b

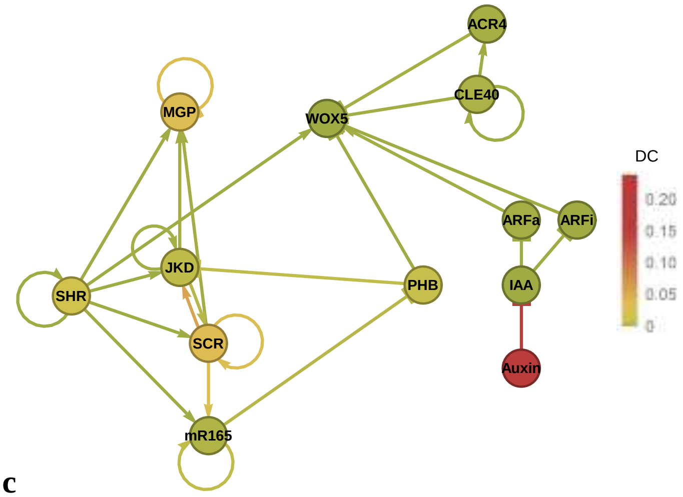

c

Supplementary Figure 5

a Total centrality, **b** value centrality and **c** dynamic centrality of nodes and connections in the fission yeast cell cycle network in a green-yellow-red colour scheme (from 0 to maximum controllability).

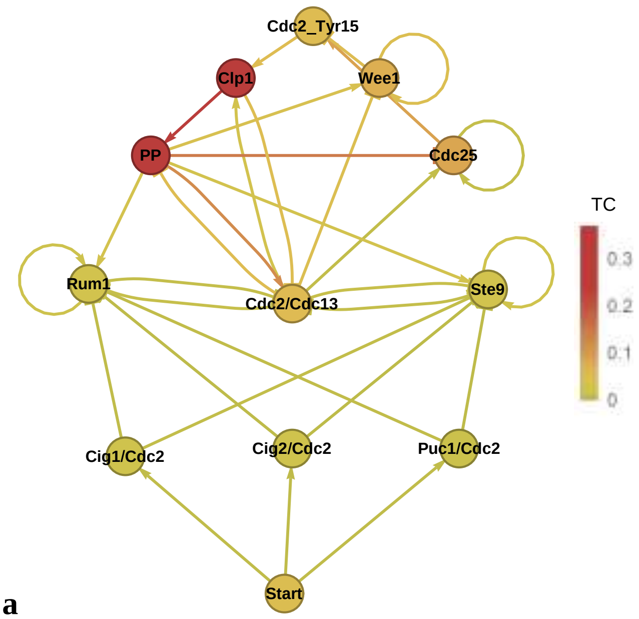

a

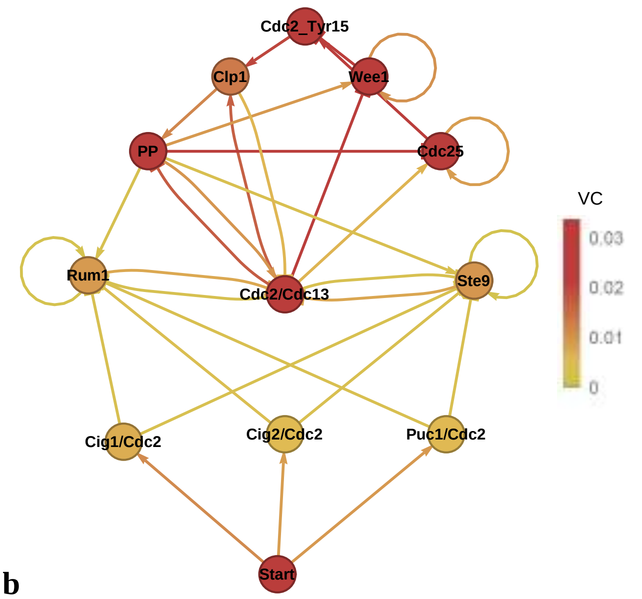

b

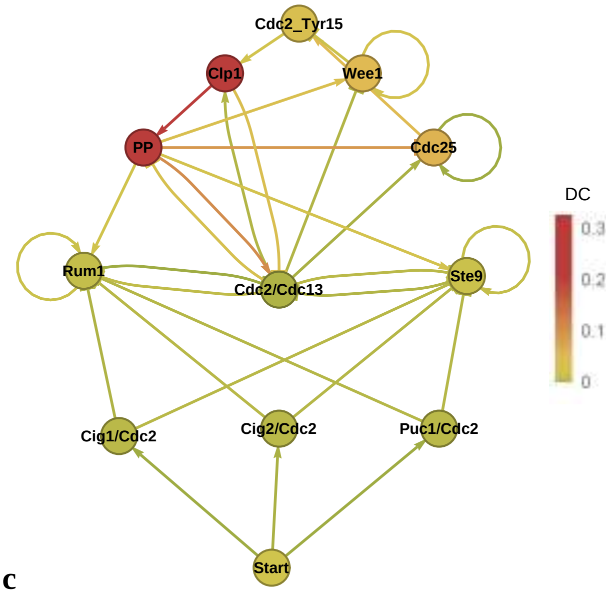

c

References

1. Karl, S. & Dandekar, T. Jimena: efficient computing and system state identification for genetic regulatory networks, *BMC Bioinformatics* **14,** 306 (2013).

2. Dixon, P., Weiner, J., Mitchell-Olds, T. & Woodley, R. Erratum to 'Bootstrapping the Gini Coefficient of Inequality', *Ecology* **69,** 1307 (1988).

3. Dixon, P. M., Weiner, J., Mitchell-Olds, T. & Woodley, R. Bootstrapping the Gini Coefficient of Inequality, *Ecology* **68,** 1548 (1987).
